# Neuro–bone tissue engineering: emerging mechanisms, potential strategies, and current challenges

**DOI:** 10.1038/s41413-023-00302-8

**Published:** 2023-12-20

**Authors:** Wenzhe Sun, Bing Ye, Siyue Chen, Lian Zeng, Hongwei Lu, Yizhou Wan, Qing Gao, Kaifang Chen, Yanzhen Qu, Bin Wu, Xiao Lv, Xiaodong Guo

**Affiliations:** 1 Department of Orthopaedics, Union Hospital, Tongji Medical College, Huazhong University of Science and Technology, Wuhan, Hubei Province China; 2 School of Basic Medicine, Tongji Medical College, Huazhong University of Science and Technology, Wuhan, Hubei Province China

**Keywords:** Bone, Neurophysiology

## Abstract

The skeleton is a highly innervated organ in which nerve fibers interact with various skeletal cells. Peripheral nerve endings release neurogenic factors and sense skeletal signals, which mediate bone metabolism and skeletal pain. In recent years, bone tissue engineering has increasingly focused on the effects of the nervous system on bone regeneration. Simultaneous regeneration of bone and nerves through the use of materials or by the enhancement of endogenous neurogenic repair signals has been proven to promote functional bone regeneration. Additionally, emerging information on the mechanisms of skeletal interoception and the central nervous system regulation of bone homeostasis provide an opportunity for advancing biomaterials. However, comprehensive reviews of this topic are lacking. Therefore, this review provides an overview of the relationship between nerves and bone regeneration, focusing on tissue engineering applications. We discuss novel regulatory mechanisms and explore innovative approaches based on nerve–bone interactions for bone regeneration. Finally, the challenges and future prospects of this field are briefly discussed.

## Introduction

The repair and reconstruction of missing or dysfunctional organs remain major challenges in the field of biomedical science.^1^ In the context of bone tissue, for small bone defects, various cell types can be mobilized to initiate endogenous repair through synergistic mechanisms.^2^ However, when the size of the bone defect exceeds a critical threshold, spontaneous healing is insufficient, necessitating additional interventions. Over the past few decades, researchers have developed diverse bioactive materials to repair bone defects, encompassing bone-mimetic ceramics, metal-based scaffolds, and natural or synthetic polymers.^3,4^ Unfortunately, these biomaterials merely replicate the macroscopic structure and mechanical characteristics of bones, with a limited ability to imitate the functional units within bones.^5^ Nevertheless, the rejuvenation of vascular and neural elements within newly formed bone structures is crucial for achieving optimal bone healing.^6^ In recent studies, angiogenesis has emerged as a critical index for assessing the efficacy of bone repair materials.^5,7^ Conversely, despite the presence of nerves in various regions of bone tissue, such as the periosteum and bone marrow, they are often overlooked in the design of bone repair materials.^1,8^

In recent years, there has been a growing focus on skeletal regulation by nerves, leading to the emergence of innovative theories and concepts. The importance of nerves in the process of regeneration was initially noted in research involving amphibian amputations and subsequently became a widely recognized phenomenon in mammals. Hence, investigators have endeavored to elucidate the mechanisms governing neuronal communication of the central and peripheral nervous systems with cells in the skeletal microenvironment, delineating their contributions to the growth, remodeling, and repair of bone.^8^ Studies on embryological anatomy have revealed that neural tissue development precedes bone tissue development during embryogenesis. This order of events occurs not only because the embryonic brain forms before the skeletal system but also because of the sequential pattern of innervation in embryonic bone development, with initial innervation occurring in the central portion of the backbone and later extending to the metaphysis until bone canals form around the neural pathways.^9^

As an early event in the process of bone repair, neural growth occurs prior to bone regeneration.^10^ A lack of nerve growth factor (NGF) signaling leads to impaired sensory innervation, delayed vascularization, decreased osteoprogenitor cells, and hindered skeletal development.^11,12^ More specifically, peptidergic nerves can directly influence bone formation by releasing neurogenic factors. These factors, including calcitonin gene-related peptide (CGRP), substance P (SP), vasoactive intestinal peptide (VIP) and neuropeptide Y (NPY), participate in bone formation and bone metabolism by interacting with receptors on bone cells.^13^ Nonosseous systems, including the vascular system and immune system, are also crucial participants in bone regeneration. Blood vessels play a vital role in providing nutrients and oxygen during bone regeneration, while nerves actively regulate bone metabolism and guide angiogenesis through the secretion of neurotransmitters and neuropeptides.^14^ Patients with neuropathy, such as congenital insensitivity to pain (CIP), are more prone to skeletal complications such as bone infections, fractures, and joint dislocations.^15^ Additionally, changes in innervation and activation are associated with orthopedic diseases such as osteoarthritis, lower back pain, and osteoporosis.^16^ The intricate interplay between nerves and bone highlights the importance of neurogenic regulation in bone metabolism and healing.

**Fig. 1:**
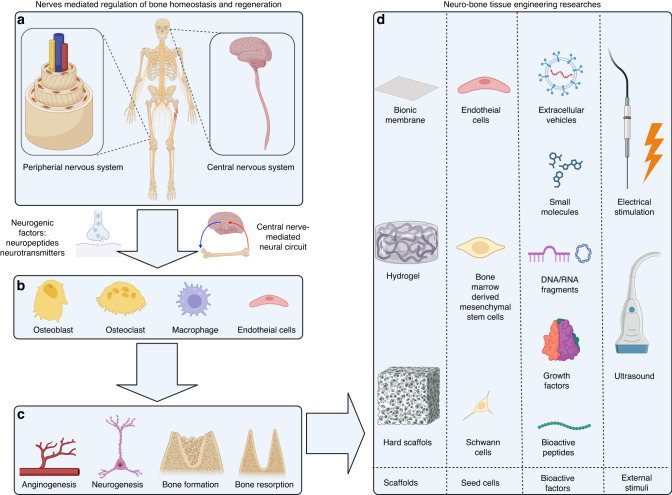
Nerve-mediated regulation of bone metabolism and neuro-bone tissue engineering research. **a** The peripheral nervous system and central nervous system regulate bone homeostasis and regeneration through neurogenic factors and neural circuits. **b**, **c** Neurogenic signals can act on various cells (osteoblasts, osteoclasts, macrophages, and endothelial cells) in bone tissue and generate bioactive responses (angiogenesis, neurogenesis, bone formation, and bone resorption), indicating their potential for use in bone tissue engineering. **d** Each component among the four elements (scaffolds, seed cells, noncell bioactive factors, external stimuli) of neuro-bone tissue engineering offers multiple choices. Created with BioRender.com

While previous reviews have touched upon the interplay between bone and the nervous system, there is a noticeable dearth of comprehensive investigations specifically examining the utilization of nerve-related components in the realm of bone tissue engineering. In this review, we first introduce the relationship between nerves and tissue regeneration and delve into the mechanisms by which nerves regulate bone regeneration. By examining the relationships between nerve regeneration, vascularization and the expression of neuropeptides following bone fracture, we emphasize the importance of the role of nerves in bone regeneration. Additionally, we introduce the concept of neuro-bone tissue engineering and present potential biomaterials for application in this approach. Finally, we propose six neuro-bone tissue engineering strategies aimed at advancing and translating the field and discuss their prospects. We expect this review to provide a reference for the study of nerve–bone crosstalk and to enrich the theoretical understanding of neuromodulatory osteogenesis, contributing to the development of bone tissue engineering and regenerative medicine (Fig. 1).

## Role of nerves in tissue regeneration

The importance of nerves in the regeneration process was initially observed through studies involving the amputation of amphibian limbs. Many organisms, such as salamanders, starfish, crabs, and lizards, possess the remarkable ability to fully regenerate certain limbs.^17,18^ In fact, flatworms can even be divided into multiple pieces, each of which has the capacity to regenerate into a complete organism.^19^ These remarkable regenerative capabilities in various organisms have sparked great interest among researchers in the field of regenerative medicine. In particular, the role of nerves in this regenerative process has garnered significant attention. In a study involving salamanders, it was observed that denervation at the brachial plexus level hindered the regeneration of upper limbs.^20^ Conversely, redirecting nerves toward the site of injury showed promise in promoting limb regeneration and even the development of supernumerary limbs.^21^ Notably, a clear connection was observed between the degree of denervation and the hindrance of limb regeneration, indicating the essential involvement of peripheral nerves in the precise control of limb regrowth.^22^

**Fig. 2:**
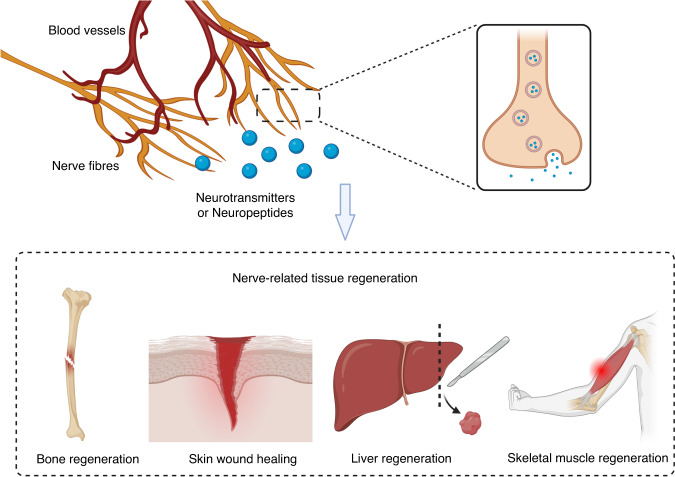
Nerve-derived neurotransmitters or neuropeptides are associated with the regeneration of bone, skin, liver, and skeletal muscle. Created with BioRender.com

Nerves are also essential for the regeneration of multiple tissues in vertebrates (Fig. 2). Since mouse toe tips exhibit impaired regeneration in the absence of local nerve innervation, nerves within bone are postulated to act as mediators of bone regeneration.^23^ Both sensory and sympathetic nerves are present in the callus and express tyrosine hydroxylase, CGRP, and SP as early markers before vascularization occurs.^10^ Nerve signals within bone can either accelerate or impede osteogenesis, maintaining the balance of bone metabolism. Peripheral nerves provide precise spatiotemporal coordination of this process during both physiological bone remodeling and repair. Furthermore, nerve-resident cells such as fibroblast-like mesenchymal cells and Schwann cells undergo transformation and proliferation upon nerve injury. Subsequently, they relocate to the injury site to facilitate bone regeneration in mice.^13^ Histomorphometry and immunohistochemical analyses showed that sensory innervation deprivation and CGRP inhibition can suppress bone remodeling and generate a proinflammatory environment.^24^ One investigation assessed how the inferior alveolar nerve affects new bone formation in rabbits and found that the absence of sensory nerves could lower the quality of newly formed bone during mandibular distraction osteogenesis.^25^ These observations suggest the involvement of peripheral nerves during bone regeneration.

The nervous system serves as the foundation of skin physiological functions and regulates wound healing and scar formation through multiple mechanisms. In clinical practice, skin wounds in patients with central nervous system injuries, such as those in the brain or spinal cord, have the potential to heal. However, chronic ulcers on the skin of diabetic patients, accompanied by peripheral neuropathy, present greater challenges for healing.^26^ Nerves actively promote skin wound healing through various mechanisms. These mechanisms include initiating neurogenic inflammatory responses, secreting neurotrophic factors to enhance blood supply to the tissues surrounding the wound, promoting the proliferation of fibroblasts and keratin-forming cells, and regulating the expression of type I and III collagen. Nerves also interact with the immune system and release neuropeptides.^27–29^ Additionally, exogenous neuropeptides show the potential to promote the healing of stubborn wounds. Conversely, denervation measures such as neuropeptide antagonists or highly selective neurotomy may help reduce scar growth.^30,31^

The restoration of liver tissue mass occurs through a combination of hepatocyte proliferation and hypertrophy in the remaining lobe after partial hepatectomy.^32^ The regenerative response to partial hepatectomy is negatively impacted by hepatic vagotomy, and the activity of the sympathetic nervous system may be suppressed during the initial stages of regeneration.^33^ In rats, hepatic vagotomy resulted in notably smaller liver-to-body weight ratios seven days after partial hepatectomy due to reduced hepatocyte proliferation.^34^ Performing hepatic vagotomy before partial hepatectomy increases mortality in mice, but this effect can be counteracted by the overexpression of FoxM1, a regulator of hepatocyte proliferation within the vagus-macrophage-hepatocyte signaling network.^35^ Sensory nerves also contribute to hepatocyte proliferation following partial hepatectomy. Neurons expressing CGRP innervate the biliary tree, and CGRP expression is heightened in rat livers after partial hepatectomy.^36^ RAMP1, a component of the CGRP receptor, is crucial for hepatocyte proliferation, as evidenced by significantly delayed regeneration in RAMP1 knockout mice following partial hepatectomy.^37^

Skeletal muscle, like other tissues, relies on vascularity and innervation for proper function. Peripheral nerves establish neuromuscular junctions (NMJs) to innervate local skeletal muscle tissue, which is essential for muscle survival, development, maturation, and contraction.^38^ When skeletal muscles are denervated, they lose contractility and undergo muscle atrophy.^39,40^ Neurotrophic factors and neurotransmitters released from neural components play a crucial role in increasing skeletal muscle cell survival and differentiation.^38,41^ Furthermore, nerves promote the regeneration of muscle blood vessels, ensuring an adequate supply of oxygen and nutrients to support muscle regeneration and repair.^42,43^

The role of nerves in tissue repair has been studied in both mammals and phylogenetically distant animals. Studies have shown that nerves regulate regeneration through the secretion of neuropeptides and neurotransmitters. Understanding these mechanisms can help identify therapeutic approaches for tissue regeneration.

## Neuromodulation of bone

### Central nervous system and bone

**Fig. 3:**
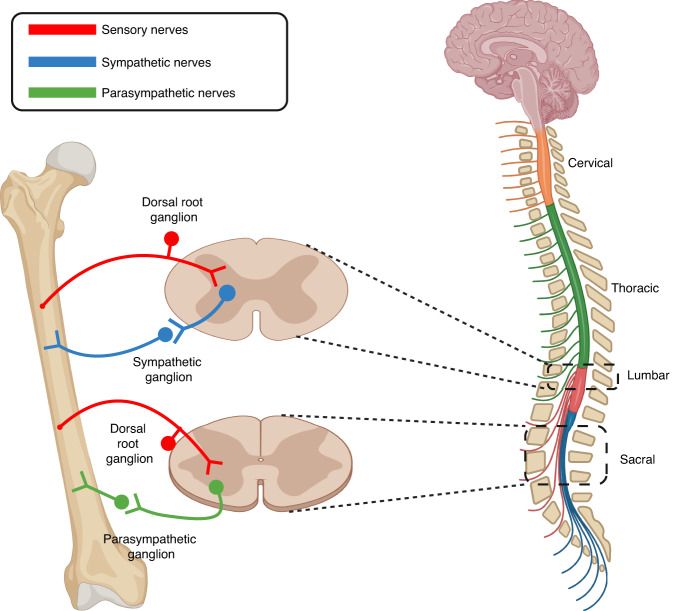
Peripheral nerves in the lower limbs originate or terminate in the lumbar spinal cord and sacral spinal cord. Both the sympathetic (blue) and parasympathetic (green) systems use two neuron relay systems to communicate with peripheral targets. The sympathetic innervation of the lower extremities originates from the lumbar spinal cord, while the parasympathetic innervation comes from the sacral spinal cord. The primary sensory neurons (red) are pseudounipolar neurons whose cell bodies are located in the dorsal root ganglion. Created by BioRender.com

The brain and spinal cord constitute the central nervous system, which analyzes and integrates information and generates coordinated responses to stimuli^44^ **(**Fig. 3**)**. The cell bodies of afferent sensory nerves are typically located in the dorsal spinal root ganglion, which connects to the central nervous system.^1^ Additionally, pseudorabies virus was detected in the sacral intermediolateral cell column and the central autonomic nucleus after inoculation into the metaphysis of the distal femur, providing evidence for the connection between autonomic nerves within bones and the central nervous system.^45,46^

An interesting phenomenon is that patients with fractures and concomitant traumatic brain injuries show increased callus formation and faster fracture healing. This suggests that brain injury may affect multiple factors during the healing process, including the release of inflammatory factors, serum leptin concentration, and the differentiation ability of mesenchymal stem cells.^47^ Injury to the spinal cord or central nervous system raises the risk of heterotopic ossification, particularly in patients who have recently undergone total hip replacement or have paraplegia.^48,49^ Additionally, spinal cord injuries caused by overstretching, trauma, or compression can result in a decrease in bone mineral density, which may be related to the upregulation of the WNT signal.^50,51^

There is a prevailing belief that skeletal system metabolism is intertwined with the endocrine system.^52^ Nevertheless, the revelation of the regulatory influence exerted by numerous neural circuits and neuropeptides has significantly increased our awareness of the involvement of the central nervous system in governing bone metabolism.^53,54^ We will describe this relationship in the following sections. Given our review’s primary focus on exploring the application of central nervous system regulation in bone tissue engineering, we advise readers to refer to earlier reviews for a broader understanding of the central nervous system’s effects on bone tissue.^53,55–57^

#### Serotonin

Throughout key brain regions, serotonin (5-HT) plays a crucial role as a neurotransmitter in learning, memory, and attention.^58^ The discovery of the presence of 5-HT receptors in major bone cell types, such as osteoblasts, osteocytes, and osteoclasts, has sparked significant interest in understanding its involvement in the regulation of bone metabolism.

In recent years, several investigations have substantiated the adverse impact of peripheral 5-HT on bone formation, specifically its role in promoting bone resorption while inhibiting bone formation.^59–61^ Yadav et al. discovered elevated serotonin levels in osteoporosis-pseudoglioma syndrome patients, which may be linked to osteoporosis symptoms. Animal studies confirmed that increased peripheral 5-HT leads to lower bone density and structural damage. Additionally, peripheral 5-HT slows the proliferation and differentiation of mouse osteoblasts, suggesting that it directly inhibits bone formation and promotes resorption via bone tissue receptors.^62,63^

In contrast to the effects of peripheral 5-HT, the central nervous system’s regulation of bone tissue by 5-HT is associated with leptin and the sympathetic nervous system. Central 5-HT can suppress sympathetic activity, thereby indirectly inhibiting bone resorption and stimulating bone formation.^64^ Conversely, leptin can inhibit central 5-HT, enhance sympathetic activity, and exert a negative influence on bone formation. Furthermore, epidemiological data show that selective serotonin reuptake inhibitors (SSRIs), which inhibit 5-HT reuptake, can reduce bone density and raise fracture risk.^65,66^

#### Semaphorin 3 A

Semaphorin 3 A (Sema 3 A), a member of the semaphorin family, is highly expressed in the hypothalamus and functions as an axon-guiding molecule, contributing to the proper development of the nervous system.^67^ Its receptor, neuropilin-1 (Nrp-1), is widely expressed in bone tissue, suggesting that Sema3A plays a role in connecting nerves and bone.

It has been demonstrated that the interaction between Sema3A and Nrp-1 can activate the Wnt/β-catenin signaling pathway in osteoblasts, inhibiting adipogenesis and suppressing the osteoclastogenesis induced by receptor activator of nuclear factor-κB ligand (RANKL). This inhibition occurs through suppression of the immunoreceptor tyrosine-based activation motif (ITAM) and RhoA signaling pathway.^68^ Studies conducted on Sema3A- and Nrp-1-deficient mice, which exhibited an osteoporotic phenotype, showed a decrease in osteoblasts and impaired bone formation, indicating that Sema3A promotes osteoblast differentiation.^69^ Similarly, mice receiving intravenous injections of Sema3A demonstrated increased bone accumulation and faster bone regeneration.^68^ Furthermore, according to Fukuda et al.,^70^ neurospecific Sema 3A-deficient mice were similar to global Sema3A knockout mice, showing a markedly low bone mass phenotype, which revealed the association between observed skeletal abnormalities and neuron-derived Sema3A.^71^ These studies suggest that Sema3A holds promise as an osteoprotective agent.

#### Brain-derived neurotrophic factor

Brain-derived neurotrophic factor (BDNF) is a versatile protein that is crucial for neural system development and is localized within a subset of dorsal root ganglia (DRG) neurons.^72^ Peripheral inflammation triggers an increase in BDNF mRNA and protein levels in TrkA^+^ sensory neurons. Subsequently, BDNF is transported to the spinal cord, where it facilitates central sensitization to pain.^73^

Both BDNF and its receptor TrkB have been identified in fracture tissues during inflammation and early bone formation, primarily in endothelial and osteoblastic cells.^72^ BDNF was found specifically in granulation tissue at the margins of woven bone and was absent in chondrocytes and mature bone.^72,74^ Osteocytes displayed strong TrkB expression, and in an in vivo study with cortical osteotomy, BDNF was observed to boost osteosclerosis. This finding implies that osteocytes may influence bone healing by regulating BDNF.^75^ BDNF has been verified to promote the proliferation and differentiation of human bone marrow mesenchymal stem cells (hBMSCs) by elevating integrin β1 expression through TrkB-mediated activation of the ERK1/2 and AKT signaling pathways.^76,77^ BDNF additionally amplifies RANKL production by hBMSCs, thereby facilitating osteoclastogenesis.^78^ These apparently contradictory impacts on bone may arise from the epigenetic control of BDNF transcription, wherein distinct physiological or pathological circumstances lead to alternative splicing and polyadenylation.^13^

### Peripheral nervous system and bone

**Fig. 4:**
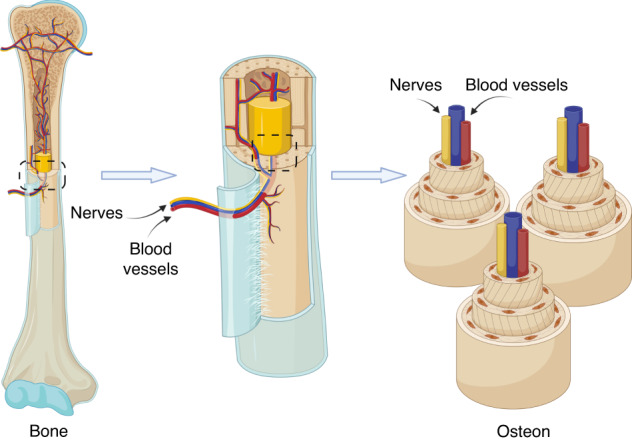
Distribution of nerves and blood vessels within the bone. Created with BioRender.com

The peripheral nerves that innervate human bones consist primarily of sensory and motor nerves, which transmit stimuli or responses through action potentials or chemical signals. These innervations are distributed throughout the periosteum, trabeculae, subchondral bone, and bone marrow^79^ **(**Fig. 4**)**. Motor nerves within the bone mainly consist of autonomic nerves, which are classified as sympathetic or parasympathetic based on their pharmacological characteristics. However, the specific density and pattern of parasympathetic nerves in the bone remain a topic of debate.^80^

Sensory nerve innervation is crucial for bone remodeling and repair.^6^ Damage to sensory innervation slows bone formation and increases bone resorption, resulting in reduced trabecular thickness and increased bone fragility.^81^ In a rat-angled fracture model, there was a higher presence of CGRP-positive innervation in the angulated concave regions than in convex regions, suggesting a greater need for sensory innervation in the concave region for bone repair.^82^ Numerous sprouted nerve fibers could be observed in the hyperplastic periosteum, callus, and fibrocartilage edge of a tibia fracture before vascular ingrowth and bone mineralization.^10,83^ In addition to its role in regulating the internal environment, the autonomic nervous system maintains bone homeostasis.^57^ Studies have shown that sympathectomized rats exhibit significantly reduced mineral content and density in the lumbar vertebrae.^84^ Mice lacking β2-adrenergic receptor expression still displayed a high bone mass phenotype after ovariectomy.^85^ Additionally, after ovariectomy, nerve density in rats decreased significantly, supporting the connection between the nervous system and bone loss following ovariectomy.^86^ Furthermore, microgravity and inner ear vestibular dysfunction-induced bone loss appear to be associated with sympathetic overactivity.^87,88^

Neurogenic factors, including CGRP, SP, VIP, and NPY, as well as neurotransmitters such as norepinephrine (NE) and acetylcholine (Ach), play a crucial role in the influence of peripheral nerves on bone.^80^ These factors bind to specific receptors present in bone tissue, affecting processes such as bone formation, bone resorption, angiogenesis, and macrophage polarization. These effects have important implications for bone homeostasis and bone regeneration. In the subsequent sections, we will provide a detailed description of these mechanisms.

#### Calcitonin gene-related peptide

CGRP is synthesized primarily in the DRGs and transported to synaptic vesicles to regulate the activity of bone cells.^89^ Within the bone, CGRP-positive nerve fibers are more densely concentrated in the periosteum, which is crucial for new bone formation, than in the trabeculae and bone marrow.^90^

Research has shown that CGRP upregulates osteogenesis-related genes and promotes the mineralization of osteogenic cells, both dose-dependent effects.^91,92^ By binding to the RAMP1-CLR complex, CGRP released from sensory nerve endings stimulates osteogenic differentiation in periosteum-derived stem cells through the cAMP-CREB pathway.^90^ Further research has substantiated that CGRP inhibits bone resorption by increasing the OPG/RANKL expression ratio in osteoblasts. Likewise, CGRP induces the intracellular accumulation of cAMP, promotes insulin-like growth Factor I (IGF-I) expression, and hinders the production of tumor necrosis factor-alpha (TNF-α) in osteoblasts, thereby promoting a favorable bone metabolic balance.^93^ Additionally, it inhibits osteoclast differentiation by suppressing the RANKL-NF-κB signaling pathway.^94,95^

Through its interaction with inflammatory factors, CGRP can augment vascular permeability and facilitate vasodilation, consequently exerting regulatory control over blood flow within blood vessels.^96^ Additionally, CGRP released from DRGs is associated with angiogenesis during osteoporotic fracture healing and critical bone defect regeneration, which greatly affects nutrient supply during fracture healing.^97,98^

Moreover, CGRP has emerged as a potent immunomodulatory factor that can interact with macrophages to promote their transformation from M1 to M2 subtypes, thereby inhibiting inflammation in local tissues and promoting tissue healing.^99,100^ Notably, this may be related to the PI3K/AKT signaling pathway and necessarily involves the assistance of other cytokines.^101,102^

#### Substance P

Similarly, SP is a neuropeptide that is widely distributed throughout the nervous system.^103^ SP-positive nerve fibers are found predominantly in regions of the skeletal system with high metabolic activity, including long bones, periosteum, joints, and epiphyseal growth plates.^104^

The impact of SP on bone remains contentious, possibly due to variations in its concentration. Recent studies have shown that high concentrations of SP (>10^−8^ mol·L^−1^) can upregulate osteogenic genes in bone marrow-derived mesenchymal stem cells (BMSCs), potentially by modulating autophagic activity and reactive oxygen species generation.^105,106^ In contrast, low concentrations of SP (<10^−8^ mol·L^−1^) inhibit the osteogenic differentiation of BMSCs and only maintain their self-proliferation.^107^ Additionally, it has been reported that SP can enhance endogenous mesenchymal stem cell recruitment during calvarial bone defect repair.^108^ On the other hand, SP has been implicated in enhanced bone resorption through the RANKL/OPG axis.^109,110^ The activation of its receptor, NK1-R, and downstream NF-κB signaling pathways may contribute to increased osteoclastogenesis.^111,112^ Noticeably, either excessive release or depletion of SP has been associated with increased numbers of osteoclasts, as observed in capsaicin-induced inactivation of sensory neurons with reduced SP expression, which leads to an osteoporosis phenotype.^113^ Hence, SP simultaneously regulates the functions of osteoblasts and osteoclasts in a dose-dependent manner.

In addition, SP has been shown to have a variety of biological functions, including angiogenesis and immune regulation. The inhibition of endogenous endopeptidase activity or exogenous SP injection has been shown to accelerate angiogenesis during wound healing, which is associated with enhancing granulocyte recruitment and activation.^114–116^ Spinal cord injury functional recovery after SP injection may be associated with the M2 polarization of macrophages.^51,110,117^

#### Neuropeptide Y

NPY, a prominent neuropeptide, is predominantly synthesized and expressed within the nervous system.^118^ In the peripheral nervous system, NPY coexists with NE in the sympathetic nerves.^119^ NPY is involved in a range of biological functions, including vasoconstriction, the regulation of appetite and energy balance.^107^ Notably, NPY also plays a pivotal role in bone metabolism and the regulation of remodeling. These effects are mediated through direct and indirect signaling via Y1 and Y2 receptors, respectively.^120^

However, the reported bone formation outcomes induced by NPY include varying and inconclusive findings. Previous studies have demonstrated that mice with genetic knockout of NPY exhibited elevated bone density, increased mineral content, and enhanced cortical bone formation.^121^ Additionally, the induction of adipogenesis by NPY and its inhibitory effect on BMSC osteogenesis have been linked to the aging process and are closely connected with the development of osteoporosis.^122^ During fracture repair, heightened NPY-positive innervation was detected on the convex side of tibial angled fractures, hinting at a possible association with diminished callus thickness on the same side.^123^ In contrast, NPY was found to promote the proliferation and differentiation of bone marrow stromal cells into osteoblasts through the activation of the Wnt/β-catenin pathway in rat experiments.^124^ Furthermore, NPY mitigates excessive stress-induced bone loss by modulating central and peripheral noradrenergic neurons through Y2 receptor mediation.^125^ NPY has also been shown to promote postfracture bone healing through Y1 receptor signaling.^126^ Additionally, global deletion of the Y1R gene hampers the initial stages of fracture repair, resulting in reduced callus volume and strength, consequently leading to delayed fracture healing.^127^

Notably, NPY also influences the expression of VEGF as well as the migration of BMSCs in vitro, suggesting that it may be involved in angiogenesis during fracture healing.^128^ Therefore, further investigation is warranted to better comprehend the intricate effects of NPY on bone homeostasis and healing.

#### Vasoactive intestinal peptide

VIP is highly prevalent in the periosteum, and nerve fibers containing VIP are broadly distributed throughout the Haversian system and within Volkmann’s canal, which extends from the diaphysis to the epiphysis.^129^ Generally, VIP coreleases alongside ACh from cholinergic nerve terminals and exerts its physiological effects by activating VPAC1 and VPAC2 receptors.^130^ VIP has emerged as a multifunctional molecule with diverse physiological functions, including its role in bone tissue homeostasis and regeneration.^129^

In vitro investigations have revealed that VIP may augment the osteogenic differentiation of BMSCs through activation of the Wnt/β-catenin signaling pathway.^131^ Moreover, a reduction in VIP levels within the bone has been noted following ovariectomy, and this decrease is associated with the development of postmenopausal osteoporosis.^132^ Further research has demonstrated that VIP can enhance osteogenic marker expression and promote bone fracture healing in mice subjected to sympathectomy.^133^ Additionally, a recent study showed that VIP can inhibit osteoclast differentiation and temporarily suppress osteoclast activity and bone resorption.^134^

Unlike some other neuropeptides, VIP exhibits notable biological activity in blood vessels, making its utilization in bone repair a promising prospect. VIP plays a role in angiogenesis in a variety of conditions, such as arthritis and tumors.^135,136^ The gradual release of VIP within the wound has a positive impact on granulation tissue growth and angiogenesis, leading to a substantial enhancement of the wound healing process.^137^ Furthermore, because VIP has a vasodilatory effect on blood vessels, it augments local tissue blood flow, ensuring an ample supply of nutrients that are crucial for wound healing.^138^ Finally, a recent study found that VIP stimulates tubular formation in endothelial cells (ECs) and enhances the expression of VEGF in BMSCs.^129^

#### Norepinephrine and acetylcholine

NE serves as the pivotal neurotransmitter in the sympathetic nervous system and exerts its function mainly through the widely expressed β‐adrenergic receptors (β‐AR) in bone tissue.^139,140^ Elevated sympathetic activity is well known to increase epinephrine levels in urine, consequently suppressing osteogenic activity. Specifically, NE inhibits hBMSC proliferation by activating β2-AR and inducing ERK1/2 and PKA phosphorylation.^141^ The impact of NE on BMSCs may contribute to impaired bone formation, possibly explaining the increased bone loss observed with glucocorticoid use.^141,142^ Additionally, NE stimulates osteocytes to produce RANKL, a crucial factor in osteoclast differentiation, ultimately enhancing osteoclast maturation.^143^ NE also directly boosts osteoclastogenesis by mediating intracellular ROS production, and the use of β-AR inhibitors such as propranolol has demonstrated corresponding inhibitory effects on this process.^144,145^ Importantly, the administration of propranolol to mice with femur fractures and posttraumatic stress disorder (PTSD) ameliorated the observed impairment of new bone formation.^146^ These research findings suggest that countering the influence of norepinephrine on bone tissue has potential as an approach for bolstering bone regeneration.

Likewise, ACh acts as the principal neurotransmitter released by cholinergic nerve fibers and exerts its biological functions by binding to nicotinic acetylcholine receptors (nAChRs) or muscarinic acetylcholine receptors (mAChRs) on cells.^147,148^ Prior studies have demonstrated that ACh stimulates osteoblast proliferation but has a limited effect on osteoblast differentiation.^45^ Ligands targeting nAChRs can dose-dependently decrease the count of osteoclasts and tartrate-resistant acid phosphatase-positive monocytes in vitro.^149^ Similarly, mice lacking the α(2)nAChR subunit exhibit elevated bone resorption and reduced bone mass.^45^ The activation of nAChR inhibits RANKL-induced Ca^2+^ oscillation, negatively regulating the osteoclastogenesis process through weakened Ca^2+^-NFATc1 signaling.^149,150^ Moreover, research results have indicated on the importance of cholinergic mechanisms during skeletal embryonic development. Experiments involving the implantation of ACh-soaked beads into chicken limb anlagen resulted in in ovo accelerated bone formation.^151,152^ Notably, it is crucial to clarify the distinct roles of mAChR subtypes within the skeletal system. A comprehensive understanding of the in vivo biological effects of ACh on bone tissue remains elusive, highlighting the need for future research.

### Skeletal interoception

Although it is generally believed that the peripheral nervous system and the central nervous system have independent effects on the skeletal system, recent research has revealed a coordinated mechanism indicating that they may jointly participate in skeletal interoception.^153^ In this mechanism, PGE2, a member of the prostaglandin family, plays a key role by binding to EP4 receptors. Essential enzymes in the biosynthesis of PGE2, include prostaglandin E2 synthase-1 (mPGES-1) and cyclooxygenase (COX).^154^ Interestingly, the use of nonsteroidal anti-inflammatory drugs (NSAIDs), which are COX enzyme inhibitors, for pain management may trigger adverse effects such as delayed fracture healing and increased risk of nonunion. This effect further supports the idea that the disruption of PGE2/EP4 signaling may hinder bone regeneration.^155^ Although PGE2 is not directly released by neurons, it can activate skeletal interoception and initiate the regulation of bone tissue metabolism and regeneration by nerves, providing a new perspective on nerve–bone interactions.

**Fig. 5:**
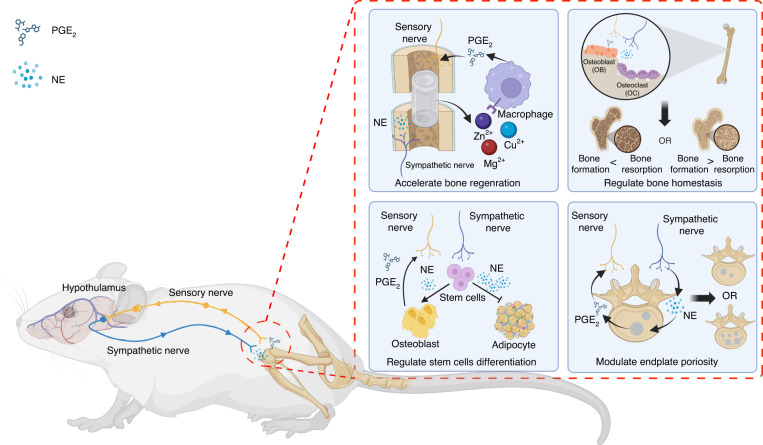
Skeletal interoception regulates bone metabolism by coordinating sensory and sympathetic nerve activity. Bone implants containing Mg^2+^, Zn^2+^, and Cu^2+^ can enhance bone regeneration by activating skeletal interoception. Activated skeletal interoception participates in maintaining bone homeostasis by regulating stem cell differentiation lineage. In bone degenerative conditions, modulating the concentration of PGE2 by celecoxib to maintain a physiological level can activate skeletal interoception, which contributes to decreased endplate porosity and to pain relief. Created with BioRender.com

Research on skeletal interoception strives to enhance our comprehension of how skeletal sensory nerves sense the state of bone tissue. The prevailing belief is that ascending signals from bone tissue reach the central nervous system, particularly the hypothalamus, via sensory nerves, dorsal root ganglia, and the superficial dorsal horn of the spinal cord (Fig. 5).^153^ Reportedly, PGE2 from osteoblasts during bone resorption can bind to EP4 receptors on sensory nerves as an ascending signal that can sense bone density and thereby modulate autonomic nerve activity via the hypothalamus.^144^ Reducing sympathetic tone through these neural circuits promotes mesenchymal stem cell transformation into osteoblasts and inhibits local adipogenic effects, which is crucial for maintaining bone mass.^156^ In aging-related disc degeneration, vertebral endplates become porous. Low-dose celecoxib, a COX-2 inhibitor, can maintain PGE2 levels, mitigating endplate porosity and pain via inflammation and skeletal interoception regulation.^157^ Certain metal ions from implants, such as Mg^2+^, Zn^2+^, and Cu^2+^, stimulate PGE2 secretion by macrophages, aiding bone regeneration through this neural circuit and revealing the role of divalent cations in healing.^158^ This discovery not only underscores the pivotal role of implants in bone repair but also emphasizes the importance of specific metal ions in orchestrating the intricate interplay between nerves and bone. It opens up new avenues for the development of more efficient implant materials and treatment strategies, exhibiting potential for positively influencing bone health and the healing process. The details of crosstalk between the brain and skeletal tissue remains a mystery.^159^ However, this interaction is vital for overall bodily equilibrium. Delving into these mechanisms is crucial, as it has the potential to yield tangible clinical advancements.

### Activity of nerves during bone fracture and regeneration

During the process of bone regeneration, nerves play an active role in responding to damage signals caused by external forces or pathological changes that disrupt the bone structure. These injury signals propagate in a retrograde direction along the proximal axon to the cell bodies, triggering the transition of nerves into a regenerative state. This transition involves the initiation of regeneration-related metabolism and the secretion of neuropeptides such as CGRP and SP around the fracture site.^97–99^ The terminals of sensory nerves are equipped with various receptors, including Nav1.7, Nav1.8, Nav1.9, TRPV1, and TRPA1, which can be activated by inflammatory mediators and growth factors. The activation of these receptors leads to sensitization and the release of additional neurotransmitters.^12,160,161^ Concurrently, the distal axon undergoes Wallerian degeneration, resulting in deterioration of the axon, myelin, and blood-nerve barrier. Schwann cells (SCs) play a role in clearing debris that contains signals impeding axonal growth, and recruited macrophages contribute to the clearance process at the site of injury.^162^ Repair SCs generate provisional channels called SC basal lamina tubes, which serve as guides for regenerating proximal axons, directing them toward the target organ for reinnervation of the bone.^163^ In summary, the primary reactions of peripheral nerves to bone fractures involve inflammation and Wallerian degeneration.

**Fig. 6:**
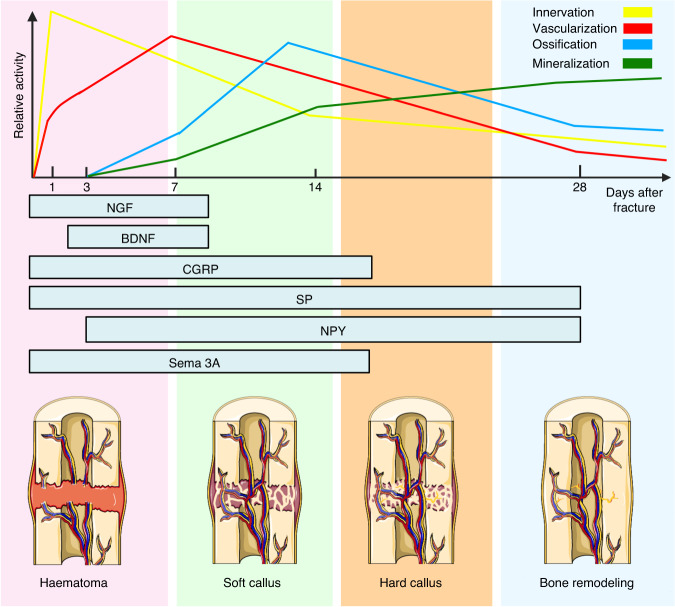
Expression of neuropeptides and related events during bone healing. The upper line chart displays the temporal sequence of events following a bone fracture. The x-axis represents the number of days after fracture, while the y-axis depicts the relative activity of different events. Neuropeptides (NGF, BDNF, CGRP, SP, NPY, and Sema3A) show a distinct distribution across the four phases (inflammatory, soft/cartilaginous callus, hard/bony callus, and remodeling) of bone healing. Created with BioRender.com

Bone regeneration is a complex process that involves the restoration of the bone’s original microarchitecture and simultaneous reinnervation. Although these processes may seem unrelated, they are intricately intertwined, as they occur concurrently during the regenerative process.^6,13^ Researchers have found that adequate innervation density is crucial for salamander regeneration, which suggests that hyperinnervation may be necessary after bone fractures.^22^ As the callus forms and matures, the regenerated nerves undergo sprouting as the bone matrix is deposited. Eventually, these nerves become restricted to the outer fibrous capsule of the hard callus after being trimmed. Reinnervation is facilitated by both CGRP-positive and TH-positive nerve fibers, with CGRP nerve fibers playing a primary role.^10^ After the completion of bone repair, the nerve fibers within the bone typically return to their normal levels. However, in cases of fracture nonunion, persistent hyperinnervation can be observed in the vicinity of the bone, periosteum, cortical bone, and bone marrow.^164^ If excessive innervation is not corrected promptly, it can lead to chronic pain-related pathological conditions. Following bone injury, various neuropeptides and neurotransmitters are expressed in the healing microenvironment, which mediates nerve regeneration and neuromodulation of bone regeneration.^10,97,98^ Notably, NGF derived from macrophages plays a critical role in skull bone regeneration by stimulating the inward growth of sensory nerves.^10^ The distribution of increased neuropeptides varies during the four classical phases of bone healing, namely, the inflammatory phase, callus formation, bone callus formation, and the remodeling phase^10,13,165–170^ (Fig. 6). The bioactive molecules generated during bone regeneration influence not only the peripheral nervous system but also the vasculature and immune system, thereby promoting bone regeneration processes.^97,98,128,161,171^

## Neuro-bone tissue engineering

While bone tissue does exhibit a natural regenerative ability that is adequate for the repair of small areas of damage, large defects resulting from traumatic injuries, degenerative diseases, congenital anomalies, or surgical tumor removal necessitate medical intervention to achieve functional restoration.^4^ At present, autologous or allogeneic bone grafts are considered the gold standard for repairing bone defects.^172^ However, the use of autologous bone grafts requires additional surgery at the tissue acquisition site.^173^ Compared to autologous bone grafts, allogeneic bone grafts are rendered nonviable through irradiation or freeze-drying, which reduces their bone inductivity and may ultimately lead to graft failure.^174,175^ While cellular bone matrices have been recently developed to enhance the bioactivity of grafts, they carry an increased risk of immune rejection and do not guarantee cell viability during preservation and implantation.^175^ At the same time, allogeneic transplantation carries inherent risks related to disease transmission, reduced structural integrity, and higher costs.^175^ Driven by pressing clinical needs, the field of bone tissue engineering has emerged and undergone rapid development in recent decades.

The bone tissue engineering research field seeks to develop materials that exceed the capabilities of bone autografts and allografts.^4^ Currently, various types of materials are available for bone tissue engineering, including natural or synthetic polymers and inorganic materials.^176–179^ Some of these biomaterials successfully mimic the structure and mechanical properties of bone. Additionally, some researchers have used a layer-by-layer design to mimic the hierarchical microstructure of bone, and such strategies have achieved some success in bone defect repair. However, few approaches have been able to mimic the functional units within bone, such as blood vessels or nerves, which has led to suboptimal outcomes.^54^

As previously mentioned, nerve fibers are widely present in bone and, to some extent, influence bone tissue regeneration, a function that has gained widespread attention in recent years. Previous research has emphasized the close interaction between bone tissue and neural tissue in various pathophysiological states and proposed potential scaffolding strategies for neuro-bone tissue engineering.^54,79,80^ In the field of neuro-bone tissue engineering, scaffolds have been ingeniously engineered to carry neural cells or growth factors. This innovative approach has facilitated the development of intricate neural networks within the scaffolds, enabling nerve-coordinated bone regeneration. Furthermore, given the influence of neuropeptides on the bone regeneration process, direct loading of these neurogenic factors or harnessing the favorable effects of the nervous system on bone regeneration is also considered a viable strategy. In this process, the nerves provide support for angiogenesis within the scaffold. Simultaneously, the intricately coordinated interaction between neuronal elements and vascular formation accelerates bone cell growth and yields outstanding regenerative outcomes.^5,8,54^ Therefore, in contrast to singular approaches to bone tissue engineering or neural tissue engineering, neuro-bone tissue engineering emphasizes the synergistic role of nerves during the bone regeneration process. New evidence suggests that neuro-bone tissue engineering has broad application prospects, but its current application status and design concepts require further elucidation, which we will discuss in the following sections.

### Scaffolds in neuro-bone tissue engineering

In bone defect repair, biomaterials serve as three-dimensional frameworks for cell attachment, growth, and differentiation. In neuro-bone tissue engineering, the scaffold material plays a crucial role in creating an environment conducive to the regeneration of both nerve and bone tissues and in facilitating communication between the two. While there are already various materials available for repairing neural and bone tissues separately, limited research has focused on constructing materials specifically for neuro-bone tissue engineering. However, it is logical to consider that materials with applications in repairing both types of tissues have potential for use in neuro-bone tissue engineering. Additionally, some materials promote bone regeneration by influencing neural activity and can be incorporated into neuro-bone tissue engineering scaffold materials. In the following sections, we will discuss these materials in more detail.

#### Polymers

Polymers are organic materials composed of atoms interconnected through covalent bonds. Both naturally derived and synthetic polymers are valuable in bone tissue engineering. Naturally derived polymers, such as collagen, gelatin, hyaluronic acid, chitosan, alginate, and silk, are produced by living organisms.^5,180–182^ They undergo degradation into carbon dioxide and water, resulting in minimal inflammatory reactions in vivo. Synthetic polymers, including polylactic acid (PLA), polyglycolic acid (PGA), and poly(lactic-co-glycolic acid) (PLGA), can degrade under physiological conditions but may release acids that induce inflammation.^5,183,184^ However, synthetic polymers offer versatility through chemical modifications and molecular alterations, allowing the customization of properties for specific applications, such as the incorporation of functional motifs to enhance cell-scaffold interactions.^185^ Table 1 summarizes examples of the applications of different materials in bone and nerve regeneration.

**Table 1:** Examples of polymers and inorganic materials applied in bone and nerve regeneration

Materials		Characteristics	Examples for nerve regeneration			Examples for bone regeneration			Refs
			Scaffold components	Morphology and state	Functions	Scaffold components	Morphology and state	Functions	
Natural polymers	Collagen/Gelatin	Naturally located within connective tissue; Possess bioactive motif for cell adhesion	Collagen; Laminin; Fibronectin; Glycosaminoglycans; PLA; PCL	Nerve conduit	Repair sciatic nerve defects	Collagen; Glycosaminoglycan; PCL	3D-printed scaffold	Enhance osteoclastogenesis, angiogenesis, and neurogenesis during bone regeneration	^364,365^
	Hyaluronic acid	Water retention ability; Biodegradability; Biocompatibility	Hyaluronic acid; Laminin	Nerve conduit	Repair sciatic nerve defect	Hyaluronic acid; Fluorenylmethyloxycarbonyl-diphenylalanine	Hydrogel	Promote osteogenic differentiation	^366,367^
	Alginate	Easy accessibility; Biodegradability; Biocompatibility	Valproic acid; Alginate; Chitosan	Hydrogel	Promote regeneration of spinal cord injury	Silk fibroin; Mesoporous bioglass; Sodium alginate	Hydrogel	Promote osteogenic differentiation of BMSC; Regulate macrophage polarization	^368,369^
	Chitosan	Biodegradability; Biocompatibility; Positively charged for electrostatic interaction with anionic molecules	Chitosan; Graphene oxide; PCL	Nerve conduit	Improve sciatic functional index and electrophysiology	Nano-Hydroxyapatite; Chitosan; Strontium ions; Polydopamine	Microspheres	Promote mesenchymal stem cells osteogenic differentiation and the endothelial cells vascularization	^370,336^
	Keratin	Ubiquitous in the epidermis and hair	Human hair keratin; Schwann cells	Artificial nerve graft	Promote nerve conductive and motor function after sciatic nerve injury; Increase the expression of nerve injury repair factors	Keratin; BMP2; Hydroxyapatite	Freeze-casting scaffold	Improve mechanical properties; Sustained rhBMP2 release; 3D cellular infiltration	^371,372^
	Silk	Biocompatibility; Biodegradability; Excellent mechanical properties	Silk; NGF; Neural stem cells	Hydrogel	Provide suitable microenvironment for spinal cord repair; Promote scarless tissue regeneration with improved functional recovery	PCL; Chitosan; Silk fibroin; PCL; plasmid	Bilayer nanofibrous mats	Enhance both osteogenesis-related gene expression and the formation of mineralized nodules	^373,374^
Synthetic polymers	PLA	Relatively poor biocompatibility; High reproducibility; Creation of acidic environment during degradation	PLA; Collagen	Electrospun film	Provide sufficient mechanical support; Create favorable microenvironment for axon regeneration	PLA; HA; Vancomycin; PLGA	3D-printed scaffold	Promote the proliferation of adipose stem cells	^375,376^
	PGA	Relatively poor biocompatibility; High reproducibility; Creation of acidic environment during degradation	PGA; Collagen	Nerve conduit	Relieve muscle atrophy; Improve the sciatic functional index	PGA; Bioactive glass; Collagen	Porous scaffolds	Promote bone regeneration in vivo	^182,184^
	PLGA	Tunable mechanical strength and degradation rate; creation of acidic environment during degradation	PLGA; PEG; erythropoietin	Thermogel	Enhance sciatic function index and withdrawal reflex after sciatic nerve injury	PLGA; HA microspheres; BMP-2;	Composite scaffold	Trigger osteogenic differentiation of BMSCs	^377,378^
	PCL	Low melting temperature, Good mechanical strength; Biocompatibility; Biodegradability	PCL; Magnesium ions; Methacrylated hyaluronic acid hydrogel	Nerve conduit filled with hydrogel	Significantly enhance axon regeneration and remyelination	PCL; FeS_2_	3D-printed scaffold	Increase neovascularization and bone regeneration in vivo	^379,380^
Inorganic materials	Hydroxyapatite	Main component of bone tissue; Bioactivity; Biocompatibility	Hydroxyapatite; Gelatin	Hydrogel membrane	Enhance neurite elongation	Hydroxyapatite; Silk; BMP2; VEGF; NGF	3D-printed scaffold	Upregulate osteoblastic associated genes; Promote neurovascular bone regeneration	^181,186^
	Tricalcium phosphate	Bioactivity; Biocompatibility; Biodegradability	β-TCP; Hydroxyapatite; Chitosan; NGF	Nerve guide conduit	Promote peripheral nerve regeneration	β-TCP; PLC	Porous scaffolds	Dynamic degradation; Regulate macrophage polarization; Promote bone regeneration in vivo	^187,188^
	Magnesium	Degradability; Biocompatibility; Excellent Young’s modulus; Abundant in natural bone tissue	Magnesium; Silk; Chitosan	Porous nerve guidance conduit	Promote the growth of damaged nerves	Magnesium; Zinc; α-TCP; Alginate; Gelatin	3D-printed scaffold	Enhance vascularization and reinnervation during bone regeneration	^381,382^

*BMP2* bone morphogenetic protein 2, *VEGF* vascular endothelial growth factor, *NGF* Nerve Growth Factor, *β-TCP* Beta-tricalcium phosphate, *PLA* polylactic acid, *PCL* Polycaprolactone, *BMSC* Bone mesenchymal stem cells, *PGA* Polyglycolic acid, *PLGA* Poly(lactic-co-glycolic acid)

#### Inorganic materials

In bone tissue engineering, inorganic materials play a significant role, particularly bioactive ceramics and metals (Table 1). Bioactive ceramics such as hydroxyapatite and calcium triphosphate possess excellent biocompatibility and osteoconductivity due to their similar composition to bone. They also provide a continuous supply of phosphorus and calcium ions, which promote biomineralization. However, pure ceramic materials alone cannot recruit cells, regulate immunity, or promote neurovascular regeneration. Hence, they are often used in combination with other bioactive materials to better fulfill the needs of neuro-bone tissue engineering.^186–191^ Metals, such as titanium and stainless steel, are commonly used in clinical metal implants. They exhibit favorable biocompatibility and mechanical properties. However, the non-biodegradable nature of these implants often necessitates a second surgery for removal. On the other hand, magnesium, which has an excellent Young’s modulus similar to that of natural cortical bone, is an important metal in bone tissue engineering. Mg-based implants not only avoid stress shielding but also contribute to immune regulation, nerve regeneration, angiogenesis, and bone repair. Notably, studies have demonstrated that magnesium can stimulate the secretion of CGRP by nerves, effectively mobilizing endogenous signals for nerve–bone repair.^97,191,192^ Consequently, magnesium holds great potential for applications in neuro-bone tissue engineering.

#### Composite materials

In neuro-bone tissue engineering, the demand for composite materials arises from a multitude of intricate factors, making the use of a single material unfeasible and thus necessitating the utilization of composites of existing materials (Table 1). First, in the context of neuro-bone tissue engineering, the consideration extends beyond the mere repair and regeneration of bone tissue to encompass the crucial promotion of neural tissue regeneration. The regeneration of these two distinct tissue types has disparate biological and mechanical prerequisites. For instance, materials must offer mechanical support for bone tissue regeneration and create a conducive environment for the growth of neural cells. Composite biomaterials offer a promising solution to meet these diverse needs. Moreover, neuro-bone tissue engineering aspires to faithfully replicate the intricate structure and functionality found in natural biological tissues, which entails scaffolds that mimic the complex structures of both bone and neural tissues while delivering essential biological functionalities. Furthermore, the successful regeneration of bone hinges on the synergistic interplay between neural and bone tissues. The advantage of composite materials resides in their capacity to facilitate favorable interactions between neural and bone tissues through the precise modulation of material combinations, thereby fostering synergistic regeneration. Last, the utilization of composite materials serves as an effective strategy to surmount the limitations inherently associated with single materials.

Polymers offer excellent biocompatibility, as natural polymers such as collagen are inherent components of biological tissues, providing innate bioinformatic guidance that enhances cell adhesion and tissue regeneration. Furthermore, synthetic polymers can be purposefully tailored, aligning them with desired biomaterial functions without altering their intrinsic properties.^193^ However, polymers may have relatively weak mechanical properties that do not necessarily suffice for the high strength demands of neuro-bone tissue engineering.^4^ In contrast, inorganic materials such as bioceramics and metals exhibit outstanding mechanical properties suitable for bone repair. They typically do not elicit immune responses and are thus well tolerated in the body.^4^ Nevertheless, some inorganic materials may lack the bioinformatics guidance needed for promoting cell adhesion and tissue regeneration, necessitating additional enhancement efforts.^194,195^ Furthermore, certain inorganic materials may induce adverse reactions in biological systems, necessitating careful screening and design.^196^

Considering these factors comprehensively, the common practice is to employ a composite materials approach by integrating polymers and inorganic materials to maximize the exploitation of their respective advantages while simultaneously mitigating their individual limitations. This integrated utilization can lead to more comprehensive performance and biocompatibility, offering the prospect of achieving significant breakthroughs in the field of neuro-bone tissue engineering. Thus, polymers and inorganic materials play complementary roles in biomedical engineering, offering increased hope and potential for tissue treatment and repair.

### Seed cells in neuro-bone tissue engineering

Seed cells are fundamental elements in tissue engineering. Ideal seed cells for bone tissue engineering should possess strong proliferative capacity, robust adaptability to the environment, and good tissue compatibility.^175^ As seed cells are the primary contributors to the biological activity of bone tissue engineering scaffolds, previous research in bone tissue engineering has primarily emphasized their osteogenic differentiation potential, often overlooking the influence of seed cells on other endogenous cells. While all types of stem cells can undergo secondary differentiation, inducing stem cells into neuronal and osteogenic lineages simultaneously is undeniably challenging. From another perspective, in the field of neuro-bone tissue engineering, all seed cells that can promote the synergistic regeneration of neural and bone tissues should be considered. To date, various seed cells, including mesenchymal stem cells, Schwann cells, and endothelial cells, have been employed in neuro-bone tissue engineering.

#### Bone marrow mesenchymal stem cells

BMSCs are a subpopulation of cells found in the mammalian bone marrow stroma. They have remarkable ability to differentiate into various cell types, including bone, cartilage, adipose, neural, and myogenic cells, making them ideal seed cells for neuro-bone tissue engineering.^197^ In theory, any stem cell capable of differentiating into either bone or nerve cells can be used, but it is challenging to simultaneously induce neural and osteogenic differentiation in the same stem cells. Therefore, BMSCs are often coimplanted with other cells, particularly SCs. Coculturing SCs and osteoblasts has been found to maintain the normal secretion of neurotrophic factors by SCs and to promote the proliferation and differentiation of osteoblasts.^198^ In a recent study, a bilayered structure was constructed, mimicking the spatial distribution of cranial bone and skull-associated nerves. This biomimetic design greatly facilitated bone regeneration by strategically distributing SCs and BMSCs.^199^ Additionally, combining sensory nerves with BMSCs on β-TCP scaffolds and implanting them into critical defect sites in rabbit femurs enhanced neovascularization and new bone formation.^200^ Studies have also shown that the BMSC-derived extracellular matrix, which contains various microenvironmental cues, such as biochemical, spatial, and biomechanical factors, provides a favorable environment for nerve regeneration.^201^ When mesenchymal stem cell-conditioned medium was loaded onto a 3D-polycaprolactone (PCL) scaffold, it effectively promoted nerve regeneration after axotomy.^202^ Overall, the regenerative effects of BMSCs are attributed to their ability to differentiate and replace damaged tissues, as well as their interaction with surrounding tissues through paracrine signaling. This creates a suitable microenvironment that jointly regulates the proliferation and differentiation processes of tissues and organs. However, achieving the codelivery of multiple seed cells in a scaffold still requires compromises in the crosstalk and interactions between the different cell types.^198^

#### Schwann cells

Schwann cells are peripheral glial cells that play a crucial role in peripheral nerve regeneration. Following nerve injury, SCs collaborate with macrophages to clear myelin debris and proliferate to form bands that support axon growth by secreting growth factors and extracellular matrix.^203^ The transplantation of SCs has been shown to enhance axon outgrowth both in vitro and in vivo.^204,205^ Moreover, SCs have been found to exert nutritional and regulatory effects on bone metabolism through complex mechanisms. In a mouse mandibular osteotomy model, the absence of SCs and their paracrine factors resulted in functional defects in mandibular skeletal stem cells, leading to a decreased rate of mandibular bone repair.^206^ These findings highlight the importance of SCs and their paracrine effects in the regenerative differentiation of skeletal stem cells. Recent studies have also demonstrated that Schwann cell-derived extracellular vesicles promote the migration, proliferation, and osteogenic differentiation of BMSCs.^207^ Researchers have successfully utilized multicellular 3D bioprinting technology to create a neural-bone construct that serves as an innervated-bone organoid. Incorporating SCs into β-calcium triphosphate tissue engineering scaffolds with BMSCs and ECs has been shown to effectively promote bone tissue regeneration through the dual effects of neurogenesis and angiogenesis.^208^ These findings indicate the promising research and application prospects of SCs in bone tissue engineering. Future studies should focus on understanding the regulatory effects of interactions between SCs and various cell types in bone tissue, including gene and protein expression as well as intercellular signal transduction. Exploiting the characteristics of SCs in tissue cell proliferation, secretion, adhesion, migration, and differentiation will facilitate the development of novel therapeutic strategies suitable for orthopedic clinical applications.

#### Endothelial cells

Bone is a highly vascularized connective tissue, and adequate vasculature is crucial for proper bone development, regeneration, and remodeling. In cases of large bone defects resulting from disease or trauma, bone grafting or bone tissue engineering approaches are necessary. However, the success of bone regeneration in both methods relies heavily on the establishment of a proper blood supply.^209^ Additionally, recent studies have highlighted the importance of crosstalk between ECs and neural cells in peripheral nerve regeneration.^210^ The blood vessels within regenerating nerves serve as pathways for Schwann cell migration, facilitating the formation of bands of Büngner that promote axonal regeneration.^211^ In coculture systems, ECs can promote the proliferation and migration of SCs, contributing to peripheral nerve regeneration.^212^ Aligned tube-like structures composed of ECs in engineered nerve constructs have been shown to enhance nerve regeneration in rat sciatic nerve models.^211^ VEGF, a crucial growth factor involved in angiogenesis regulation, can be secreted by ECs, and hydrogels containing VEGF have a synergistic effect with BDNF on peripheral nerve regeneration.^210^ Therefore, ECs also hold promise in bone tissue engineering, as they can simultaneously enhance nerve and bone regeneration. Qin et al. successfully produced a cell-laden scaffold with proangiogenic properties using 3D bioprinting technology. This was accomplished by incorporating vascular endothelial cells into a bioink based on Li-Mg-Si (LMS) bioceramics. The bioactive ions released from LMS, in conjunction with the capacity of vascular cells to secrete advantageous cytokines for neural cells, supported the differentiation of neural and osteogenic cells within the scaffolds.^213^

### Acellular bioactive factors

Effective osteogenesis or neurogenesis necessitates the incorporation of acellular biological components, including growth factors, peptides, small molecules, DNA/RNA fragments, or exosomes, into scaffolds. These elements serve to provide supplementary stimuli that enhance neurogenesis or osteogenesis. While numerous investigations have delved into the impacts of different factors on bone or nerve tissues, there remains a dearth of acellular factors that can concurrently foster the regeneration of nerves and bones. Within this section, we delineate acellular elements that independently facilitate the regeneration of bone and nerves. Moreover, when these factors are employed in tandem, they exhibit substantial potential within the field of neuro-bone tissue engineering.

#### Growth factors

Biological reactions are initiated by growth factors, which are soluble signaling proteins secreted by cells. They bind to cell surface receptors and regulate intracellular signaling cascades, playing crucial roles in various cellular processes.^214,215^

In bone formation, several growth factors are involved, including bone morphogenetic proteins (BMPs), transforming growth factor-β (TGF-β), platelet-derived growth factor (PDGF), fibroblast growth factor (FGF), and insulin-like growth factor (IGF), among which BMP is widely studied.^216^ BMP can stimulate pluripotent cells such as BMSCs to proliferate and differentiate into chondrocytes and osteoblasts, promoting new bone formation.^217^ Thus, growth factors, when encapsulated within scaffolds, can be used to treat fractures effectively.^181,218,219^ Additionally, BMP has been shown to stimulate angiogenesis, further contributing to bone regeneration.^220^ Currently, BMP-2, 3, 4, 5, and 7 are the main osteogenic factors known, with BMP-2 and BMP-7 being the most extensively studied and approved by the FDA for bone regeneration.^221^

Nerve growth factors (NGF), brain-derived neurotrophic factors (BDNF), glial-derived neurotrophic factors (GDNF), and neurotrophic factors-3 and -4 (NT-3, NT-4) are crucial for neuronal growth and survival.^222^ During nerve regeneration, these neurotrophic factors play a role in enhancing stem cell differentiation, recruitment, and remyelination processes.^223^ The gradient of neurotrophic factors produced by nerve terminals after trauma exerts both stimulatory and inhibitory effects on nerve repair.^224^ Incorporating neurotrophic factors into neural conduits to promote nerve regeneration has been explored in several studies, through approaches involving either injection or modification approaches.^225–227^

Compared to conventional bone tissue engineering scaffolds, this approach, particularly with the incorporation of nerve growth factors, produces scaffolds with neuroattractive capability. The incorporation of growth factors within the scaffold offers potential for achieving the synergistic regeneration of both bone and neural components. Nevertheless, the utilization of growth factors in neuro-bone tissue engineering faces challenges such as elevated costs, unstable biological activity, and potential adverse effects.^228^ The high-dosage application of growth factors in attempting to enhance therapeutic effectiveness at the fracture site may precipitate inflammation, tumor formation, and ectopic bone development in unforeseen regions, especially when employed in tandem.^229,230^

#### Bioactive peptides

Incorporating bioactive peptides with active protein motifs into scaffolds offers a cost-effective alternative to the use of growth factors. Short peptide sequences derived from natural or synthetic materials stimulate cell surface receptors and regulate signaling pathways.

Peptides derived from growth factors such as osteopontin, BMP-2, and BMP-7 can be incorporated into scaffolds to promote osteogenesis in vivo.^231–233^ For instance, a modified PA66 polymer scaffold containing BMP-2-derived peptide and QK (a VEGF mimetic peptide) has successfully repaired severe femoral fractures in SD rats.^234^ Treatment with bone-forming peptide-1 (BFP-1), a peptide sequence from BMP-7, upregulates the expression of osteogenic genes in BMSCs, leading to greater bone formation in vivo than that elicited by the original growth factors.^232^ Peptides derived from the extracellular matrix that promote cell migration and proliferation are also effective tools in bone tissue engineering. Dos Santos et al. developed a growth factor-free hydrogel consisting of elastin-like polypeptides (ELPs), poly(ethylene glycol) (PEG), and a range of concentrations of the adhesion peptide IKVAV. This hydrogel promotes angiogenesis and innervation during bone repair.^185^

Bioactive peptides are also useful in nerve tissue engineering. Nerve conduits containing bioactive peptides that mimic the functions of BDNF and VEGF have been used to repair sciatic nerve defects in rats. These peptides enhance nerve regeneration and promote vascular penetration, leading to nerve regeneration and functional recovery.^180,235^ In peripheral nerves, laminin plays a crucial role in Schwann cell migration and axon extension. A self-assembling peptide nanofiber hydrogel, dual-functionalized with the laminin-derived motif IKVAV and the BDNF-mimetic peptide epitope RGI, provides a three-dimensional microenvironment for SCs and neurites, which was successfully used to bridge a 10-mm sciatic nerve defect.^163^ The Arg-Gly-Asp (RGD) sequence, one of the most potent ligands in natural extracellular proteins, enhances the adhesion and proliferation of SCs and promotes the axon outgrowth of dorsal root ganglions when incorporated into PCL scaffolds.^236^

Compared with proteins, peptides have unique advantages. Their chemical structure can be precisely controlled, they are more tolerant to temperature and pH changes, and they are more convenient to use. To date, bioactive peptides have been widely used in the field of nerve regeneration and bone regeneration, but how to use these peptides in neuro-bone tissue engineering has not been studied. This approach provides new opportunities for future research and application.

#### Small molecules

Small molecules, referring to natural or synthetic compounds with low molecular weights, exert therapeutic effects by modulating cellular functions.^237^ These molecules, characterized by their uncharged or hydrophobic properties, can penetrate the phospholipid bilayer of the cellular membrane.^238^

Osteoinductive molecules have been found to influence various signaling pathways within cells, including BMP signaling, the hedgehog (Hh) pathway, and Wnt/beta-catenin or cyclic adenosine monophosphate/protein kinase A (cAMP/PKA) signaling pathways.^237^ For instance, the small molecule SVAK-12 has been shown to potentiate the osteogenic differentiation of myoblasts induced by BMP-2 in vitro. Moreover, the percutaneous injection of SVAK-12 has been found to accelerate rat femoral fracture callus formation.^239,240^ The effects of statins, cholesterol-lowering drugs, on bone formation have also been observed both in vitro and in vivo over several years.^241^ Recent research has demonstrated the potential of aspirin, a common drug for relieving minor aches, pains, and fevers, in promoting bone regeneration.^242^

Although the use of small molecules in peripheral nerve regeneration is limited, they show promise in enhancing neuronal viability, supporting axonal outgrowth, and influencing the neuronal differentiation of stem cells.^5^ For example, GSK-J1, LDN193189, SB431542, CHIR99021, and P7C3-A20 have documented potential to induce the neural differentiation of stem cells.^243,244^ Additionally, small molecules such as LM22B-10, FK506, BT13, and *Lycium barbarum* polysaccharides (LBP) have been shown to enhance nerve regeneration both in vivo and in vitro.^245–248^ On the other hand, drugs that compete with neurotransmitters that negatively affect osteogenesis can also be beneficial for bone regeneration. Propranolol, an adrenergic β-receptor blocker, competes with NE released by overactive sympathetic nerves, effectively blocking its negative effects on osteogenic differentiation. Similarly, nifedipine, a calcium channel blocker, promotes bone formation by inhibiting the release of NE.^145,249,250^

Small-molecule drugs have garnered significant interest as potential substitutes for growth factors due to their ease of synthesis, regulatory effects, and broad applicability. They can facilitate tissue regeneration by modulating biological pathways, although their use in nerve regeneration requires further investigation and clinical validation. Nonetheless, this offers a promising avenue for collaborative repair in nerve–bone tissue engineering.

#### DNA/RNA fragments

Although both growth factors and bioactive peptides can stimulate bone and nerve regeneration, the exogenous introduction of these factors often results in decreased activity, a short half-life, and the need for repeated high-dose administration. To address these challenges, the implantation of DNA/RNA fragments into scaffolds can be utilized as an alternative approach to avoid the use of high concentrations of growth factors.

Delivery of DNA fragments can be achieved through viral or nonviral vectors to target cells and modify their gene expression. However, the permanent integration of gene sequences into the host genome using viral vectors such as retroviral and lentiviral vectors is undesirable due to potential adverse effects.^251^ Nonviral approaches are considered safer, as they have lower immunogenicity and exert only transient effects on gene expression compared to viral delivery. Loading plasmid DNA (pDNA) into cationic scaffolds or nanoparticles appears to be a potential solution to the difficulty of transporting negatively charged pDNA across cell membranes.^252^ Introducing BMP-2 plasmid DNA into bone defects can provide a continuous source of growth factors to upregulate bone formation-related genes.^253^ VEGF plasmids and NGF plasmids loaded onto 3D-printed porous bone scaffolds can effectively replace seed cells and growth factors, inducing minimal immune response and promoting vascularized bone and nerve regeneration.^254,255^ The injection of a plasmid encoding fibroblast growth factor 2 (FGF2) around peripheral nerves has also shown potential in facilitating the regeneration of the sciatic nerve.^256^ Furthermore, the delivery of plasmids encoding miR-200c using CaCO_3_ nanoparticles resulted in the successful repair of craniofacial bone defects.^257^

In addition to DNA sequences, RNA delivery can also be employed to hinder the translation of mRNA sequences that impede bone or nerve regeneration. MicroRNAs (miRNAs) are small, naturally occurring noncoding RNAs that regulate gene expression by modulating posttranscriptional processes. Numerous miRNAs have been identified as key regulators of neurobiological processes, including neurite outgrowth, synaptogenesis, and neural plasticity. Injecting a thermoresponsive hydrogel for the codelivery of microRNA-222 and aspirin (miR222/MSN/ASP hydrogel) promoted the neuronal differentiation of BMSCs and functioned synergistically with aspirin to enhance osteogenesis.^183^ Loading miR-29b onto a 3D-printed scaffold with sustained release capabilities also demonstrated the promotion of bone regeneration, similar to the effects of other bioactive molecules in bone tissue engineering.^258^ Similarly, siRNA has been utilized to silence specific genes that inhibit osteocyte formation or facilitate osteoclast formation with high specificity.^259–261^ The use of siRNA targeting fidgetin-like 2 (FL2), a negative regulator of axon regeneration, can significantly enhance functional nerve recovery.^262^

Integrating DNA/RNA fragments into scaffolds to facilitate the synergistic regeneration of both bone and nerves holds significant promise and warrants further exploration.

#### Extracellular vesicles

Almost all types of cells secrete extracellular vesicles (EVs) that contain multiple signaling factors, such as proteins, lipids, mRNAs, miRNAs, and other noncoding RNAs, enabling communication between neighboring or distant cells during the natural healing process.^263^ These ingenious carriers, specifically those derived from SCs, MSCs, and macrophages, which are associated with bone and nerve regeneration, have been investigated for therapeutic use. The lipid bilayer protects the cargoes within EVs from degradation within the extracellular environment.^264^

In the context of bone regeneration, incorporating Schwann cell-derived exosomes into hydrogels as a multifunctional neuromodulation platform can orchestrate the bone microenvironment through immunomodulation, angiogenesis, and osteogenesis.^265^ Similarly, Su et al. developed exosome@aptamer (EA) constructs by coupling aptamers targeting phosphatidylserine (PS) with repair Schwann cell exosomes, which were loaded onto the surface of electrospun fibers to create a biomimetic periosteum with the ability to promote nerve and bone regeneration.^266^ Additionally, combining Schwann cell-derived exosomes with porous Ti6Al4V scaffolds or hydrogels has been shown to effectively improve the efficacy of scaffolds for bone defect repair in vivo.^267,268^ Similarly, the incorporation of MSC-derived EVs into scaffolds has been demonstrated to enhance bone repair in rodent calvarial bone defects and promote osteogenesis even at ectopic sites.^189,269^ Porous 3D PLA scaffolds coated with MSC-Exo have shown immunoregulatory and osteogenic effects, reducing proinflammatory marker expression and promoting osteogenic differentiation.^270^ Furthermore, macrophage-derived exosomes can promote the differentiation of BMSCs toward an osteoblastic fate through microRNA-21a-5p.^271^ Biomimetic mineralized collagen can mediate endogenous bone regeneration by recruiting MSCs and increasing the secretion of extracellular vesicles by macrophages.^272^

Extracellular vesicles secreted by various cells have also been shown to promote nerve regeneration. For example, SCs communicate with neighboring axons during regenerative processes via exosomes, which significantly increase axonal regeneration in vitro and enhance regeneration after sciatic nerve injury in vivo.^273^ BMSC-derived exosomes have been found to promote the regeneration of peripheral nerves through the miRNA-mediated regulation of regeneration-related genes in a dose-dependent manner.^274,275^ Exosomes from macrophages, which contain active NADPH oxidase 2 (NOX2) complexes, can be taken up by DRGs via endocytosis during nerve regeneration, playing a necessary role in neurite growth by mediating ROS signaling.^276^

As EVs have been employed in both bone and nerve repair, researchers are presently directing their efforts toward the identification of EVs that promote synergistic healing in bone and intrabone nerves.

### Strategies in neuro-bone tissue engineering

**Fig. 7:**
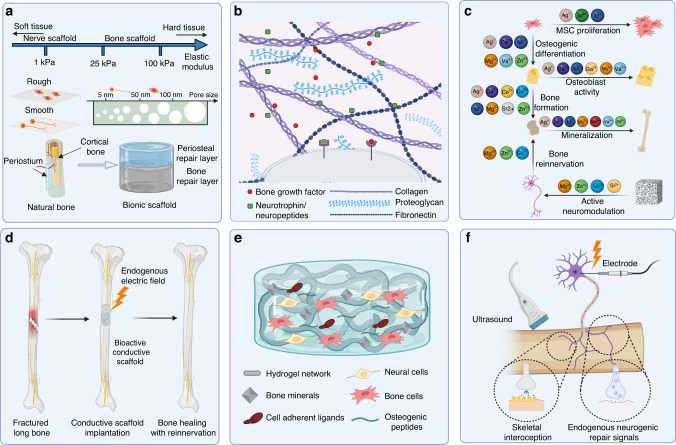
Schematic representation of neuro-bone tissue engineering strategies. **a** Regulate cell differentiation by controlling the surface morphology and structures of scaffolds; (**b**) regulate cellular behavior by modifying scaffolds with neural growth factor/neuropeptides. **c** Incorporate bioactive ions that can influence cellular behavior into scaffolds. **d** Scaffolds containing electroactive nanoparticles. **e** Coculture systems mimic the crosstalk between different cells during bone regeneration. **f** External field stimulation activates endogenous neurogenic repair signals, including skeletal interoception (left) and neuropeptide secretion (right). Created with BioRender.com

As a potential branch of bone tissue engineering, neuro-bone tissue engineering combines neural tissue engineering and bone tissue engineering, emphasizing the importance of neural repair in bone regeneration and the crosstalk between bone and nerves during this process. Several strategies have been explored (Table 2, Fig. 7), including (i) controlling the surface morphology and structures of scaffolds; (ii) incorporating neurotrophic factors and neuropeptides into the scaffolds; (iii) using ion-doped materials to enhance nerve regeneration; (iv) adding conductive additives to facilitate neural signaling; (v) constructing coculturing cell systems to promote neural and bone cell interactions; and (vi) applying external field stimuli to stimulate neural growth and response.

**Table 2:** Summary of strategies in neuro-bone tissue engineering

Strategies	Outcomes	Schematic representation	Reference
Scaffold architecture	Cellular adhesion, proliferation, and differentiation	Fig. 7a.	^285,296^
Growth factor/Neuropeptides	Long-term release with growth factor initiates nerve and bone regeneration/replaces the role of the nerve during bone regeneration	Fig. 7b.	^181,383^
Biomaterials incorporating bioactive ions	Active endogenous neuronal repair signal; Promotes bone and nerve regeneration	Fig. 7c.	^153,192^
Conductive additives	Activation of different genes related to neurogenesis and osteogenesis	Fig. 7d.	^191,321^
Coculture systems	Neuronal cells enhance osteogenic differentiation; Bone cells support the survival of neuronal cells	Fig. 7e.	^347,340^
External field stimulation	Active endogenous neuronal repair signal	Fig. 7f.	^98,384,385^

#### Surface morphology/micro-nanostructures

In the field of tissue engineering, the use of microstructured scaffolds has attracted significant attention for promoting bone and nerve regeneration.^277^ Researchers have focused on mimicking complex anatomical morphologies and incorporating desired physical properties into scaffold designs.^278^ The stiffness, roughness, and porosity of bone materials have been found to influence the proliferation and neuronal differentiation of stem cells^1^ (Fig. 7a).

Notably, the neural differentiation and osteogenic differentiation of stem cells differ, and some processes are more favored on softer matrices.^279–281^ Even subtle differences in stiffness can lead to the differentiation of neural stem cells into different types of neurons, such as neurons, oligodendrocytes, and astrocytes.^282,283^ For instance, when the stiffness of the substrate material is below 500 Pa, neural stem cells tend to differentiate into neurons, while stiffer materials promote the differentiation of astrocytes or oligodendrocytes.^284^ However, it is important to note that excessively soft materials may hinder neural cell differentiation.^282^ Therefore, determining the optimal stiffness level for bone biomaterials is crucial to enhance the growth and activity of both bone and neural cells in engineered bone biomaterials.

Neuronal cells possess the ability to sense surface topography and exhibit diverse responses to different levels of roughness and micropatterns.^285^ When human neuroblastoma cells are cultured on gold substrates, the cell viability and spreading ability decrease significantly as the surface roughness increases from flat to an average roughness of approximately 100 nm.^285^ This decrease can be attributed to the increased hydrophilicity of rough surfaces, which reduces the adsorption of hydrophobic neuronal cell adhesion proteins.^286^ Moreover, neurons grown on rough substrates exhibit disrupted polarity of the actin cytoskeleton, nuclear condensation, and impaired functionality.^285^ Recent research has provided evidence that oriented nanofibers can replicate the topographical signals of fibronectin, enabling the precise manipulation of cell alignment and phenotypic manifestation. Scaffolds composed of silk fibroin nanofibers and designed to mimic the structure of the extracellular matrix not only promote the directional growth of neurons but also support the growth, migration, and organization of endothelial cells.^287–289^ Additionally, uniform longitudinally oriented microchannels have been found to support nerve growth,^290^ and ring-shaped arrays of nanopillars can attract nearby nerves to grow along the arrays.^291^ Li et al. pioneered the use of neodymium-doped whitlockite (Nd@WH) integrated into a double-layer poly(caprolactone) nanofiber membrane with a surface-oriented structure. The micropattern of the inner layer-oriented structure provides sufficient space for cell arrangement, bone formation, neurogenesis, and angiogenesis.^292^ Thus, precise control over the nanoscale roughness and arrangement of bone biomaterials is critical for regulating the growth of neuronal cells in the bone matrix.

Another crucial factor influencing the axonal growth of neuronal cells is the porous structure of biomaterials. Porous substrates provide increased surface area, making them more likely to bind with neurogenic proteins. Studies have shown that DRG neurons are more likely to develop axons when cultured on mesoporous substrates with pores approximately 300 nm in size than when cultured on flat surfaces.^293^ Similarly, neuroblastoma cells exhibit enhanced spreading morphology and cell density on nanoporous silicon substrates, with further improvement observed as the pore size decreases from 20 nm to 5 nm.^293,294^ However, there is no significant difference in the axonal growth of DRG neurons compared to that on a flat surface when the pore size increases to the micrometer range.^293^

Due to the heterogeneous distribution of blood vessels and nerves within bone tissue, natural bone exhibits inherent heterogeneity that is challenging to replicate. Simulating natural structures at the macroscale provides a feasible approach to meet the regenerative needs of multiple tissues. For example, allograft healing can be accelerated by incorporating matrix metalloproteinase (MMP)-degradable hydrogel-modified constructs, which mimic the natural periosteally mediated healing process by supporting endogenous cell migration and neurovascular network formation.^295^ Similarly, printing a tree-like bioceramic scaffold can create, through the precise programming of blade intervals and divergence angles, a spatial microenvironment that facilitates multicellular crosstalk and innervated bone regeneration.^296^ Haversian system-mimicking 3D scaffolds have been shown to induce osteogenic, angiogenic, and neurogenic differentiation in vitro while also accelerating the ingrowth of blood vessels and new bone formation in vivo.^297^ Notably, silk fibroin nanofiber scaffolds that simulate the extracellular matrix structure can induce the directional growth of neurons and facilitate the growth, migration, and arrangement of endothelial cells.^287–289^

#### Modification with neurotrophic factors/neuropeptides

Numerous studies have extensively investigated the crucial role of reinnervation in bone regeneration by delivering neuronal growth factors, with a particular focus on NGF and BDNF **(**Table 3**)**. Moreover, the application of a coaxial nanofibrous scaffold containing NGF has been found to create a neurotrophic microenvironment that accelerates the neural differentiation of stem cells, thereby enhancing the process of osteogenesis.^298^ During this process, regenerated nerves induced by sustained NGF can act as mediators in the formation of ossification centers through the NGF-TrkA signaling pathway. These sensory nerves also exhibit a regulatory effect on excessive bone formation induced by BMP-2.^299^ Previous investigations have demonstrated that the delivery of BDNF by collagen sponges or hyaluronic acid hydrogels resulted in increased bone formation compared to the control groups.^300–302^ Therefore, the loading of various neurotrophic factors and growth factors into scaffolds for bone tissue engineering represents an effective method for treating osteonecrosis and large segmental bone defects (Fig. 7b). Nevertheless, it is important to acknowledge that these bioactive factors exhibit poor pharmacokinetic properties and are susceptible to protein degradation, thus limiting their potential as therapeutic agents.

**Table 3:** Summary of recent studies on neurotrophic factors/neuropeptides modified scaffolds

Bioactive factors	Raw materials	Models/cell	Function	Ref.
NGF/VEGF/BMP2	Silk	In vitro study; hMSCs	Upregulate genes associated with osteoblastic differentiation	^181^
NGF	Chondroitin sulfate; Hydroxyapatite	In vitro and vivo study; BMSCs; Beagle dogs	Change the morphology of bone tissues and nerve fiber in the mandible of Beagle dogs; Upregulate ontogenetic and neurogenic differentiation-related genes.	^386^
NGF	PCL	In vivo study; ICR mouse	Promote physiological innervation, resulting in the growth of innervated tooth tissue	^387^
NGF	Mesoporous bioactive glass; Osteocalcin-osteopontin-biglycan; Silk fibroin	In vitro and in vivo study; BMSCs; Mouse calvarial bone defect model	Accelerate bone regeneration upon neurogenesis; Enhance neural differentiation of BMSCs	^298^
NGF/VEGF	Whitlockite; Neodymium; PCL	In vitro and in vivo study; RAECs, BMSCs and RSCs; Rat skull defect model	Promote bone regeneration with neurovascular network formation	^292^
NGF	Titanium; Chondroitin sulfate; Hydroxyapatite	In vitro study; BMSCs	Promote the differentiation of BMSCs into osteoblast and neural cells	^388^
BDNF	β-TCP	In vitro and in vivo study; hBMSCs; Mouse subcutaneous transplantation	Promote hBMSC osteogenesis and neurogenesis in vitro and in vivo	^76^
BDNF	Bone cement; Mesoporous bioactive glass	In vitro and in vivo study; Mouse osteotomy model; Murine MSCs	Promote bone fracture healing	^389^
BDNF	Hyaluronic acid	In vitro and in vivo study; Human periodontal ligament cells; Periodontal tissue defect model in dogs	Upregulate the expression of bone-related proteins; Promote alveolar bone formation	^390^
CGRP	Hyaluronic acid hydrogel	In vitro and in vivo study; BMSCs; Rat cranial defect model	High-capacity loading of CGRP in hydrogels; Long-term sustained release of CGRP; Sustained promotion of bone regeneration via CGRP	^383^
CGRP	Bio-Oss	In vitro study; Primary osteoblasts	Upregulate proliferation rate, osteogenesis-related genes, and number of mineralization nodules	^391^
CGRP	Virally transfected BMSC; Collagen scaffold	In vitro and in vivo study; BMSC; Rat cranial defect model	Upregulate osteogenesis-related genes; Inhibit osteoclastogenesis; New bone formation and mineralization in defect area	^392,393^
CGRP	HA/SA hydrogel scaffold	In vitro and in vivo study; BMSCs/Beagle skull defect model	Enhance cell adhesion and proliferation; Upregulate number of mineralization nodules; New bone formation at defect area	^304^
CGRP	Chitosan-Sr-calcium phosphate cement	In vitro study; HUVECs	Enhance cell proliferation; Good mechanical properties	^394^
CGRP	PLGA porous microspheres	In vitro and in vivo study; BMSCs	Protect stem cell function in the inflammatory microenvironment; Promote alveolar bone regeneration in the periodontitis microenvironment	^395^
CGRP/SP	Gelatin microspheres	In vivo study; Rabbit osteoporotic bone defect model	Promote osteogenesis in a dose-dependent manner; Increase the trabecular number and decrease the degree of trabecular bone separation	^396^
Sema 3 A	Ti	In vitro study; Human BMSCs	Simultaneously promote osteoblast differentiation through the synergistic activation of Wnt5A and BMP signaling pathways; Control various stages of osseointegration	^397^
VIP	GelMA	In vitro and in vivo study; EA.hy926 endothelial cells, BMSCs; Rat cranial defect model	Induce in vitro osteogenesis and angiogenesis differentiation; Facilitate bone repair through activation of the Wnt/β-catenin signaling pathway	^131^
SP	PLGA	In vitro and in vivo study; hMSCs; Rat cranial defect model	Increase stem cell recruitment; Increase bone volume at bone defect area	^398^
SP	Chitin-PLGA-calcium sulfate hydrogel	In vitro and in vivo study; hADSCs; Rat cranial bone defect model	Upregulate osteogenic gene expression; Increase stem cell migration	^399^
SP	Ti	In vitro and in vivo study; MSCs; Rat femoral defect model	Improve stem cells migration capability; Enhance secretion of matrix metalloproteinases (MMP2, MMP9); Promoting bone regeneration	^400^
SP	HA; CS; Gel	In vitro and in vivo study; BMSCs; Rabbit knee subchondral defect model	Facilitate spreading and proliferation of stem cells; Induce osteogenic differentiation and extracellular matrix mineralization; Promote subchondral bone regeneration	^401^
SP/BMP-2	Heparin-conjugated fibrin gel	In vitro and in vivo study; MSCs; Rat cranial defect model	Recruit endogenous stem cells; Promote bone regeneration in vivo	^402^
SP	CPC	In vivo study; Rabbit alveolar bone defect model	Enhance osteoconductivity, inductivity and osteogenic ability	^403^
SP/BMP-2	Ti; GO	In vitro and in vivo study; MSCs; Mouse cranial defect model	Enhance the migration of MSCs and new bone formation	^404^
SP	PLA; β-TCP; KLD12	In vitro and in vivo study; MSCs; Rat cranial defect model	Enhance the migration of stem cells; Promote bone tissue regeneration	^405^
SP	PLGA; Gelatin nanofibers	In vitro and in vivo study; BMSC; Rat immediate implant placement model	Increase stem cell transmigration and osteogenic differentiation with reduced bone resorption	^406^

*HA* hydroxyapatite, *SA* sodium alginate, *Sr* strontium, *HUVECs* human umbilical vein endothelial cells, *BMMs* bone marrow monocytes, *hMSCs* human mesenchymal stem cells, *hADSCs* human adipose-derived mesenchymal cells, *RSCs* rat Schwann cells, *RAECs* rat aortic endothelial cells, *Ti* titanium, *PVA* polyvinyl alcohol, *GelMA* gelatin methacrylate, *PLGA* poly(lactic-co-glycolic acid), *MMP2* metalloproteinase 2, *MMP9* metalloproteinase 9, *CS* chitosan, *Gel* gelatin, *CPC* calcium phosphate bone cement, *GO* graphene oxide, *PLA* polylactic acid, *β-TCP* β-calcium triphosphate

It is widely recognized that neurogenic factors, including neurotransmitters and neuropeptides, play crucial roles in mediating the interaction between nerves and bone during the process of bone regeneration. Consequently, incorporating neurogenic factors into modified materials can mimic the positive effects of nerves on bone regeneration **(**Table 3**)**. Osteogenesis-related peptides, in comparison to various macromolecular growth factors, offer several advantages, such as high efficiency, ease of synthesis, low cost, and convenient implantation in the extracellular matrix. These peptides promote cell adhesion and accelerate mineralization.^303^ Furthermore, neuropeptides possess low toxicity, high biological activity, and high specificity, making them highly suitable for clinical translation.^304^ Among neuropeptides, CGRP is widely recognized for its anabolic properties, making it a favorable candidate for introduction into bone regeneration systems to temporarily replace the function of nerves during the early stages of bone healing. However, bone regeneration is a long-term process that necessitates the continuous involvement of growth factors at each stage.^2^ Therefore, the design of bioactive scaffolds revolves around maintaining the controlled release of highly active peptides. Alternatively, efforts can be made to integrate transgenic cells into the scaffold to establish a stable source of neuropeptides. While the stable release of neuropeptides is crucial for promoting osteogenesis, the cell-free strategy appears to be more practical and feasible for application and development than the use of scaffolds containing transgenic cells, as the cell-free approach effectively circumvents immune rejection and ethical concerns.^5^ Apart from CGRP, other neuropeptides, such as VIP, SP, and Sema 3 A, have shown potential for application in bone tissue engineering. While the introduction of exogenous neurogenic factors has been shown to enhance the repair of bone defects, it is important to note that their biological effects are difficult to control due to the complex humoral environment and the widespread expression of receptors. Inaccurate and continuous delivery may lead to unexpected side effects. Therefore, future bioactive materials are expected to dynamically mimic the biological effects of nerves on bone during the bone healing process.

#### Ion-doped materials

The incorporation of bioactive ions into materials has shown promising results in enhancing neurogenic and osteogenic markers and activating neuromodulation in bone. The role of metal ions such as magnesium ion (Mg^2+^), zinc ion (Zn^2+^), and copper ion (Cu^2+^) in bone growth and remodeling has been recognized since the late 1990s.^305,306^ Over the past few decades, research has revealed the regulatory effects of cations on osteogenesis, osteoclastogenesis, angiogenesis, and immune responses.^307,308^ However, the involvement of the nervous system in the formation of new bone induced by metal ions has only recently gained attention.^153^ Different ions play crucial roles in various biochemical functions that are essential for different stages of bone regeneration. Their presence and influence help maintain a delicate balance between bone and neural cells (Fig. 7c). Among metal ions, magnesium has shown good bone regeneration and nerve regeneration capabilities in recent studies. As a commonly occurring element in nature, magnesium is abundant in the human body.^309^ Biodegradable magnesium implants exhibit excellent biocompatibility and bioactivity compared to other synthetic polymers.^310^ Recent studies have revealed that Mg^2+^ released from implants can promote the secretion of CGRP from sensory nerve endings within the bone.^90,97,311,312^ Additionally, Mg^2+^, Zn^2+^, and Cu^2+^ were found to activate skeletal interoception by modulating macrophage activity.^158^ Zn^2+^ and Cu^2+^ ions were also found to play crucial roles in modulating the biological activity of neurotrophic factors, which are important for nerve survival and regeneration. Zn^2+^ treatment in neuronal cell cultures has been associated with increased binding to BDNF and with enhanced proliferation, while the presence of Cu^2+^ synergistically affects nerve cell activity with NGF.^222^ Apart from metal ions, other inorganic ions also play a role in nerve-induced bone regeneration. For instance, silicon, an essential element for the human body, is necessary for bone homeostasis.^313^ Silicate glass has been used as a bioactive material due to its positive effects on osteoblasts, osteoclasts, and endothelial cells.^314^ However, a recent study provided new insight into silicate-based material-induced bone regeneration by triggering the release of Sema3A from DRG, which promoted rat femoral defect regeneration.^315^ Current bone substitute materials primarily offer osteoconductive healing properties, and many advanced tissue engineering strategies are not yet practical for everyday clinical use. The comprehensive examination of the physiological mechanisms of various ions and their impact on bone tissue regeneration indicates that incorporating certain ions into existing bone substitutes can potentially modify inflammation, the immune response, bone regeneration, and nerve regeneration. The utilization and combination of ions with existing biomaterials are influenced by various factors. The concentration of released metal ions has been shown to be critical for the process of bone formation. Therefore, it is advantageous to have these ions near the implanted biomaterial, facilitating bone regeneration directly at the implant site.

#### Electroactive nanoparticles

Numerous physiological functions are linked to endogenous electric fields (EFs). These functions encompass the development and organization of tissues, as well as their regeneration after injuries.^316^ Given the sensitivity of bone lineage cells and neural cells to electrical stimulation, the employment of electroactive materials could prove beneficial in facilitating the repair of both bone and nerve tissue^317^(Fig. 7d).

Over a century has passed since the first synthesis of black phosphorus (BP), which is recognized as the most stable allotrope of phosphorus.^318^ BP nanomaterials possess exceptional optical and mechanical properties, along with electrical conductivity, biocompatibility, and biodegradability, making them highly suitable for various biomedical applications.^319^ By incorporating BP into a biomimetic periosteum or hydrogel, the regeneration of nerves and bones could be stimulated, creating a favorable microenvironment for tissue repair.^191,192,320^ Through the application of electrical stimuli to MSCs cultured on BP@PDA hydrogels, significant improvements in cell migration and neural differentiation within 3D scaffolds have been observed.^321^ Furthermore, the continuous release of phosphorus ions from black phosphorus during degradation actively promotes bone mineralization and the osteogenic differentiation of mesenchymal stem cells.^322,323^

Polypyrrole (PPy), an organic polymer, is formed through the oxidative polymerization of pyrrole.^324^ Because of its excellent conductivity and antioxidation properties, PPy shows significant potential for applications in tissue engineering. The modification of scaffolds with PPy has been found to improve their mechanical properties, electrical conductivity, osteoconductivity, and drug delivery capabilities and to exert a positive effect on the growth and osteogenic differentiation of bone marrow stem cells.^325,326^ Furthermore, hydrogels incorporating PPy have been reported to exhibit beneficial bioactivity, electrical conductivity, and antioxidant properties, which help to create a neuroprotective and neuroinducible 3D environment for encapsulated BMSCs, thereby accelerating recovery from spinal cord injury.^327,328^

Polyaniline (PAni), a semiflexible rod polymer, demonstrates outstanding conductivity and serves as an organic semiconductor.^329^ Consequently, PLA scaffolds incorporating well-distributed PAni can offer an optimal environment for the growth and differentiation of BMSCs due to their conductive properties.^325^ Furthermore, coating medical titanium sheets with PAni can synergistically enhance cell proliferation and osteogenesis through electrical stimulation.^330^ Through the complexation of gold nanoparticles with PAni, reinforced nanocomposites can be delivered intracellularly and induce the differentiation of stem cells into neural cell lineages under electrical stimulation using an electroporator.^331^ Additionally, composite hydrogels containing PAni-modified carboxymethyl chitosan, when incorporated within a nerve conduit, significantly promoted sciatic nerve regeneration and Schwann cell proliferation compared to hollow chitosan conduits.^332^

Carbon-based materials such as graphene and carbon nanotubes are commonly used as conductive additives in bone and nerve tissue engineering due to their favorable mechanical properties, lack of cytotoxicity toward osteoblasts, and intrinsic antibacterial activity.^333^ The incorporation of reduced graphene oxide (rGO) into hydrogels increased the electrical conductivity of scaffolds, enhanced the osteogenic and neurogenic differentiation of loaded cells, and accelerated bone regeneration by modulating the diabetic inflammatory microenvironment.^334,335^ Similarly, scaffolds or nerve conduits fabricated by combining PLC and carbon nanotubes have been applied to support the ingrowth of subchondral bone and consequently the repair of 10 mm sciatic nerve defects in rats.^336–338^

Overall, scaffolds based on conductive materials offer the potential to harness the benefits of biological electric fields in bone and nerve tissue while also providing a substrate that promotes faster cell proliferation than that achieved with conventional synthetic materials. However, further research is necessary to explore the long-term capabilities of implanted conductive and electroactive bone scaffolds.

#### Cell coculture systems

Physiological tissues consist of multicellular systems comprising different cell types that interact with each other to facilitate viability, proliferation, and development. In recent years, tissue engineering has been moving toward cocultures, as they offer a more physiologically relevant representation of natural tissues, both physically and biologically.^339^ Compared to traditional monolayer cell culture, coculture systems closely mimic the in vivo environment, allowing better observation of cell‒cell interactions, where cells serve as stimulus sources for providing desired signals to other cell types^340^ (Fig. 7e).

Coculturing neural cells with pluripotent stem cells has shown positive effects on neurogenesis, offering an alternative strategy for reinnervation. The introduction of cocultured SCs and adipose-derived stem cells (ADSCs) as seed cells into a silk fibroin (SF)/collagen scaffold to construct a tissue-engineered nerve conduit has demonstrated improved regenerative microenvironments and accelerated nerve regeneration.^341,342^ Combining stem cells with ECs in coculture not only promotes DRG axon growth but also enhances neuronal orientation, potentially reducing the risk of axonal tangling and inhibiting neurofibroma formation.^343^ Coculturing SCs and neural stem cells (NSCs) in laminin-chitosan-poly-lactic-co-glycolic acid (laminin-chitosan-PLGA) nerve conduits has shown potential in promoting injured nerve regeneration in rats.^344^

In the context of bone regeneration, coculturing ECs with osteoblasts is often employed to induce capillary formation, while ECs similarly affect the phenotypic expression and proliferation of osteoblasts.^345^ Cocultures of BMSCs and ECs have been reported to enhance the expression of osteogenic markers and improve the proliferation of stem cells.^346^ Furthermore, extracellular matrices derived from coculture systems of mesenchymal stem cells (MSCs) and human umbilical vein endothelial cells (HUVECs) have shown significant enhancements in cell proliferation compared to that achieved on scaffolds made of PCL alone while maintaining similar physical and mechanical properties.^347^ When preosteoblasts are cocultured with neural progenitor cells in a 3D nanocomposite hydrogel incorporating whitlockite, both the neural and osteogenic activities of the coculture system are enhanced.^348^

Stem cells offer remarkable versatility in tissue engineering, allowing the creation of complex tissues and organs with enhanced regenerative potential. However, ethical concerns, regulatory restrictions, immune rejection risks, and potential tumorigenicity pose significant challenges.^349^ In contrast, the integration of multiple cell types in tissue engineering holds promise for replicating natural tissue interactions and enhancing functionality but entails complexities in managing diverse cell populations and in obtaining regulatory approval.^339^ However, the increasing popularity of coculture systems in tissue engineering highlights the importance of addressing challenges such as appropriate cell selection, the optimization of cell ratios and numbers, and the refinement of culture media to significantly impact experimental outcomes.^350^ Furthermore, ensuring the survival of these seed cells under hypoxic conditions in vivo before complete vascularization is established remains crucial for successful tissue engineering endeavors.^175^

#### External field stimuli

The nervous system can respond to external stimulation, leading to changes in cellular behavior that can be leveraged for therapeutic purposes. Current research focuses on initiating endogenous neurogenic repair signals. One widely recognized anabolic neuropeptide, CGRP, has shown promise for enhancing bone repair when its expression is enhanced in the microenvironment. Typically, the release of CGRP is increased in response to heat, low pH, and TRPV1 agonists.^351,352^ However, these microenvironmental conditions can impair normal bone healing, making it impractical to translate these strategies directly. As alternatives, strategies such as electrical stimulation and low-intensity pulsed ultrasound (LIPUS) have been developed.^2^ These approaches aim to stimulate the nervous system and elicit beneficial effects on bone repair without the need for extreme microenvironmental conditions.

Electrical stimulation (ES) is a widely researched physical therapy modality that facilitates the repair and regeneration of damaged tissues, including bone, muscle, skin, and nerve.^353^ However, the parameters and strategies of electrical stimulation in neuro-bone tissue engineering may be different from those used for bone or nerve regeneration alone. In previous studies, electric or electromagnetic fields were applied directly to the fracture site to modulate local biological functions involved in the healing process.^354^ For bone regeneration, researchers usually use specific electrical stimulation parameters, such as a frequency of 15 hertz, an electric field intensity of 200 mV·mm^−1^, and a treatment time in the range of 2–8 h.^353^ To optimize neural tissue regeneration, the parameter settings for electrical stimulation are aimed at regulating the growth and signal transduction of nerve cells. For example, primary mouse hippocampal neurons and PC-12 cells cultivated on a scaffold with optimal electrical stimulation (100-150 mV·cm^−1^ for 1 h daily) exhibited augmented growth of both neurites and microfibers.^355^ However, electrical stimulation strategies in neuro-bone tissue engineering mainly focus on stimulating nerve cells to regulate the synthesis of specific neuropeptides, such as CGRP. It was reported that applying a maximum stimulation voltage that did not cause significant contraction of the innervated muscle (250 mV, 90 μs, 150 Hz, 61.3 μA) enhanced CGRP expression in DRG neurons.^356^ The implantation of electrodes (10 V, 500 μs, and 10 Hz) into the lumbar DRG was found to improve innervation of the area of femoral fracture in rats, stimulate CGRP biosynthesis and release, and ultimately accelerate fracture healing.^98^ These studies demonstrated that a wide range of electrical stimulation parameters can be used to stimulate CGRP^+^ neurons. The use of the above electrical stimulation parameters and strategies holds promise for the future development of implants with built-in electric fields that can both promote bone and nerve regeneration through electric fields and deliver electrical stimulation to the DRG to promote CGRP-mediated bone regeneration through neuromodulation. Thus, these findings provide valuable insights into the therapeutic potential of neuro-bone tissue engineering.

Low-intensity pulsed ultrasound (LIPUS) is a commonly used physical therapy method that has shown potential for promoting fracture healing^357^ (Fig. 7f). Unlike high-intensity ultrasound energy, LIPUS is nonthermal and nondestructive, making it a promising approach for bone tissue repair and regeneration.^358^ Recent studies have demonstrated that LIPUS can effectively enhance Schwann cell proliferation and axonal remyelination, thereby promoting nerve regeneration ^304,403^. Moreover, LIPUS has been shown to enhance CGRP-positive sensory innervation, which contributes to osteogenesis, as observed in an animal model of spinal fusion.^359^ However, in a rat model of tibial fracture after sciatic nerve resection, LIPUS did not promote fracture healing.^360^ These findings suggest that the positive effects of LIPUS on bone regeneration may be mediated by promoting CGRP secretion from sensory nerve fibers. Additionally, LIPUS has been found to increase the expression of cyclooxygenase 2 (COX-2), a key enzyme involved in the synthesis of PGE2, in osteoblasts. Elevated levels of PGE2 in the local microenvironment can activate skeletal interoception, thereby accelerating bone regeneration through neuronal pathways.^361–363^

## Summary and future prospects

In this review, we delve into the influence of the nervous system on tissue regeneration from lower to higher species. We begin by providing an overview of the anatomical aspects of the nervous system within skeletal tissue and delve into the regulatory mechanisms relevant to skeletal diseases. Our focus is on addressing critical bone defect regeneration. Specifically, we emphasize the neuroendocrine and signaling functions of nerve fibers within bone tissue and their important role in fostering osteogenesis through intercellular communication. Then, we elaborated on the concept of neuro-bone tissue engineering and its basic elements, including scaffold materials, seed cells and acellular active factors, important design criteria and the latest achievements in developing neuro-bone tissue engineering materials. Finally, we combined the factors involved in neuro-bone tissue engineering to obtain six feasible strategies. In conclusion, nerve fibers enhance bone regeneration by providing neuropeptides that regulate angiogenesis, osteogenic differentiation, and immunity, and accordingly, critical bone defects can be effectively repaired by promoting the synergistic regeneration of nerve and bone or by mobilizing endogenous neurogenic repair signals.

While significant advances have been made in developing organic and inorganic materials that closely emulate the physicochemical characteristics of bone, the field of neuro-bone tissue engineering grafts is still in its nascent phase. A major obstacle involves establishing an ideal environment that facilitates the differentiation and proliferation of both nerve and bone cells within bone implant materials.

Given the highly vascularized and innervated nature of human bone, it is crucial for bone implant materials to emulate the microenvironment of bone tissue to enhance fracture healing. Recognizing the crucial role of nerves in promoting bone healing has revealed the need for deeper insights into the mechanisms underlying the interactions between nerve fibers and different cell types within skeletal tissue. Consequently, this comprehensive review aims to provide a holistic understanding of the anatomy and functional aspects of nerves in bone tissue, with the goal of advancing the field of neuro-bone tissue engineering and facilitating its clinical translation.
